# Colors and Things

**DOI:** 10.1177/2041669520958431

**Published:** 2020-11-05

**Authors:** Jan Koenderink, Andrea van Doorn, Karl Gegenfurtner

**Affiliations:** Department of Experimental Psychology, Justus Liebig University Giessen, Giessen, Germany; EECS Department, University of California at Berkeley, California, United States; Department of Experimental Psychology, Justus Liebig University Giessen, Giessen, Germany

**Keywords:** color, natural image statistics, object recognition, segmentation, surfaces/materials

## Abstract

How many colors are there? Quoted numbers range from ten million to a dozen. Are colors object properties? Opinions range all the way from *of course they are* to *no, colors are just mental paint*. These questions are ill-posed. We submit that the way to tackle such questions is to adopt a biological approach, based on the evolutionary past of hominins. Hunter-gatherers in tundra or savannah environments have various, mutually distinct uses for color. Color differences aid in segmenting the visual field, whereas color qualia aid in recognizing objects. Classical psychophysics targets the former, but mostly ignores the latter, whereas experimental phenomenology, for instance in color naming, is relevant for recognition. Ecological factors, not anatomical/physiological ones, limit the validity of qualia as distinguishing signs. Spectral databases for varieties of daylight and object reflectance factors allow one to model this. The two questions are really one. A valid question that may replace both is *how many distinguishing signs does color vision offer in the hominin Umwelt?* The answer turns out to be *about a thousand*. The reason is that colors are formally not object properties but pragmatically are useful distinguishing signs.

It was upon a Sommers shynie day,

When Titan faire his beames did display,

In a fresh fountaine, farre from all mens vew,

She bath’d her brest, the boyling heat t’allay;

She bath’d with roses red, and violets blew,

And all the sweetest flowres, that in the forrest grew.

        Sir Edmund Spenser The Faerie Queene (1590)

        Book Three, Canto vi, Stanza 6 (our italics).

## Introduction

In the aforementioned verse, the author silently assumes that *colors are object properties*. The aim of this article is to explore the validity of this folk wisdom. The answer turns out to be implicate.

Colorimetry was created by Maxwell (1855) and von Helmholtz (1855, 1867). It is amazingly successful in putting a formal structure on the possibilities of discrimination of radiant spectral power distribution by a majority of the (human) population.

Remarkably, the formal structure is almost perfectly linear, indicating that it is due to elementary physical processes rather than complicated (and most likely nonlinear) physiological, or even psychological ones. Indeed, the theory is rooted in the physical process of the absorption of photons by three distinct pigments. No neural transformations or psychological processes are involved at that stage. So any properties decided at the earliest stage do not depend on processes at higher stages of the nervous system.

The formalism^1^ involves a linear operator^2^ (to be determined empirically) acting on the space of radiant power spectra (Centore, 2017; Koenderink, 2010a). The radiant power spectra are parameterized as vectors in an infinitely dimensional Hausdorff space (Arkhangelskii & Pontryagin, 1990), the continuous parameter being wavelength.^3^ When two radiant spectra map on the same element, they are equivalent, meaning that they can be mutually substituted for each other without visible effects (Grassmann, 1853). This puts an equivalence structure, a partition, on the domain of the operator.^4^ It is the complete formalism of colorimetry. Although formally trivial, it has remarkable predictive power.

Formally, this implies that being mutually equivalent implies differing by an element of the null-space of the linear operator. This null-space is empirically of codimension three (the generic case, covering over 95% of the population), a fact known as *trichromacy*. It is a fact about the generic *human* condition.^5^ One encounters a variety of codimensions throughout the animal kingdom. Thus, except for three degrees of freedom, humans are blind to the infinities of spectral compositions as these occur in nature.

One defines a *color* as an equivalence class (Brualdi, 2004) of mutually equivalent radiant spectra. The elements of the *space of colors* then are these equivalence classes. By elementary linear geometry, these are mutually parallel copies of the null-space (Koenderink, 2010a). Color space is itself a linear space of dimension three.

Both Maxwell and Helmholtz perfectly understood this; it was axiomatized by Grassmann (1853) in the mid-19th century.

Going beyond this formalism was first attempted by von Helmholtz (1867, 1891, 1892a, 1892b). As is only to be expected, almost any such an excursion away from colorimetry proper involves nontrivial physiology or phenomenology. Consequently, *color science* is a vastly more complicated area than colorimetry proper. In this article, we draw only on basic colorimetry and physics, thus ignoring most of mainstream color science. We believe the results to be relevant for phenomenology too, because the structure of the Umwelt, together with the lifestyle, must drive the evolution of awareness.

Perhaps the best known, basic colorimetric structure is the locus of colors defined by monochromatic beams. It is a convex cone^6^ with an open sector and can conveniently be represented by the cut with some well-chosen plane. This intersection is a convex arc with the topological structure of a horseshoe, that is, a circle with a gap.^7^ This was empirically discovered by Maxwell (who called it a “hook”), and later Helmholtz.^8^ In this article, we mainly use an extended construction, specifically targeted at object colors, which is essentially due to Schrödinger (1920).

For practical purposes, one may use the Commission Internationale de l'Éclairage (cie) xyz color matching functions as a numerical representation of the linear operator for the generic human observer (CIE, 1932; Smith & Guild, 1931–1932). For pure colorimetry, any nonsingular linear transformation of it serves equally well.^9^ The cie system^10^ has the advantage of familiarity.^11^ We use it to set up a framework that is better adapted to the case of object colors. The latter formalism is fully equivalent to the cie one but better adapted because of a close relation between spectral reflectances (object properties) and (colorimetric) colors. It is closely similar to the system of generic red–green–blue (rgb) display colors and therefore more intuitive (Koenderink, 2018a).

## 

### Problems With the Colorimetric Formalism

The formalism sketched earlier is indeed perfectly capable to achieve what it was set up for. It allows one to predict whether two radiant spectra will be equivalent to the generic human observer. That is all it can do.

The formalism has led to various counterintuitive predictions, and the formalism yields no handle on phenomenology (Albertazzi, 2013; Albertazzi et al., 2015; Hering,1905–1911; Kuehni, 2004; Munsell, 1905, 1912; Quiller, 1989). A singular exception is the approach by Wilhelm Ostwald (1917, 1919). In Ostwald’s system, colors and canonical spectral reflectance functions exist on equal footing. This is an excellent idea, unfortunately largely ignored today. We will use a similar technique here, although our choice of canonical spectra differs from that of Ostwald.

A perhaps counterintuitive prediction of basic colorimetry is that it allows one to prove that *color is not an object property*. Thus, “Roses are Red, Violets are Blue,” which associates colors with objects, is (formally) nonsense. Spenser’s verse is mere artistic fancy. We formally prove this in Appendix C. One might not initially understand the theorem when formulated in terms of colorimetry proper, for there would be no mention of “white light” and “hues,” and there would be no names for qualia such as “red” or “blue.” There would just be illuminating beams I,I′,I″,⋯, spectral reflectance factors R,R′,R″,⋯, and colors c,c′,c″,…. Then, there would be possible or impossible relations holding between these. Pragmatically, the formalism is only relevant in the context of an interpretation.

An interesting interpretation of one formal theorem is that colors (in the phenomenological sense) are not object properties (in the sense of physics). It is indeed possible to design artificial roses, violets, and two radiant sources such that these (fake) roses are red, and these (fake) violets are blue as seen under one source (of fake daylight), whereas the same roses are blue, and the same violets are red under the other source (of another fake daylight). This pertains, even though a (real) daisy would look the same under either fake source, that is, “white,” as it looks under real daylight. Because it is thus visually obvious (the daisy is proof!) that both artificial sources provide *white light*, the rose and violet color change appears *magical* and implies that colors are not object properties. The formal proof is constructive, thus beyond doubt.

Notice that this is a purely colorimetric/physical fact. It simply exploits the human *blind spots* (usually known as *metamerism*) in the optical processing of spectral compositions.

Other problems with basic colorimetry have to do with the nature of *achromatic* (lack of any particular *hue*) radiation. There is simply no way to handle this colorimetrically, as this *does* refer to phenomenology. This is very annoying. One tends to assume (silently referring to phenomenology) that the monochromatic beams have mutually distinct hues.^12^ But then, Brouwer’s (1912) fixed point theorem forces the existence of a point inside the spectrum locus that is hueless.^13^

It is even more pressing for the object colors, because phenomenologically the most vivid object colors lie on a topological (full!) circle.^14^ Thus, there really *has* to be a colorless object color. Fortunately, in the context of object colors, one may call physics to the rescue.

### The White and Black Objects

Although not strictly colorimetry, there certainly exists a principled way to define *white* without reference to phenomenology. This necessarily involves object color. We are not aware of any such arguments that might help define achromatic beams. In the latter case, all one might do is to arbitrarily point out a certain beam as achromatic by decree.^15^

We skip a thorough discussion of phenomenology here. It should be understood that *object color* also implies *being seen* as an object (or part of an object). This is by no means guaranteed. It requires an *ecologically valid* context.

An obvious definition of a white object would require it to at least scatter all incident radiation *without any spectrally selective process*. That is obviously a necessary condition for achromaticity and one that singularly applies to object colors.

The requirement is by no means sufficient. It would include perfect mirrors and diffraction gratings. A mirror has no color, and the diffraction grating has all colors. A mirror may take on any color, depending on what is reflected in it. A diffraction grating shows different colors as you view it from different directions.

An additional requirement is that a white object should scatter the same beam to the eye, irrespective of where the eye is with respect to the object. This invariance might be taken as a definition of *visual object*. If this seems odd, then remember that a mirror is a physical object, but by no means a unique visual object. It lacks a color. A mirror may appear in any color, depending on what it incidentally happens to reflect.

Technically, this implies that the white object has to be *a Lambertian surface of unit spectral albedo*. This definition does in no way refer to particular materials. It is very generic.

Although our “white object” is a purely formal one, it is also necessary to have white reference objects in radiometric laboratory practice. In practice, white chalk or blotting paper come close. Pressed baryte (${\text{BaSO}}_{4}$) powder or a “smoked on” magnesium oxide (MgO; Tellex & Waldron, 1955) has been used as a white standard in laboratories for decades. Yet another issue involves “white references” in a scene. We cannot go into that complicated issue here. A wide range of possibilities has been indicated (Koenderink, 2010a). One possible candidate is the maximum of the red, green, and blue coordinates over the whole scene.

The definition of the white object is simply *physics*. It does not refer to phenomenology. Thus, one may well ask whether such an object actually *appears white* at all? The answer is that it sometimes does, sometimes not. In this article, we stick to settings in which it usually does (to be explained later).

A black object is even simpler to define than the white object. It is a surface that does not scatter any radiation in any direction. Thus, an aperture that opens into infinite empty space (say the clear night sky between stars, “outer space”) would be perfect. Again, this definition is simply *physics*. It does in no way refer to particular materials. It is generic and unique. In the laboratory, one has to be satisfied with material implementations such as black velvet.^16^ Not perfect, but often good enough.^17^

Thus, the realm of object colors readily admits of a number of concepts—such as black and white—that greatly increase the use of colorimetric methods. It imposes fundamental constraints that the colorimetry of radiant beams lacks.

One might attempt to define an achromatic beam phenomenologically as a beam that makes a white object *appear* white. But this will not work. In practice, one finds that white objects appear white under a wide range of colorimetrically distinct beams. People tend to use contextual information. This makes excellent biological sense. Consequently, colors as appearances do not uniquely correspond to colorimetric coordinates. Indeed, from an ontological perspective, colors as appearance and colors as colorimetric objects are mutually distinct entities.

### Colorimetry in the Presence of a Standard Illuminant

Historically, sunlight or average daylight have been intuitively taken as *standard* (Goethe, 1810; Newton, 1704; Ostwald, 1917, 1919; Quiller, 1989; Runge, 1810; Schopenhauer, 1816) by which one means *colorless*, or *achromatic*. From a biological perspective, this makes sense. Why is water tasteless? Obviously because you were born with water in your mouth. The same argument applies to daylight.

Hominins evolved in savannah or tundra environments (Gibbons, 2006). We still have a visual system that is optimized for a hunter-gatherer lifestyle in such environments. Average daylight would be the norm. *Colors* would be distinguishing marks pertaining to generic objects (ripe berries are red, unripe ones green, say). Thus, perhaps the major importance of color is in locating key objects for bare survival (Gegenfurtner & Rieger, 2000; Witzel & Gegenfurtner, 2018).

A colorimetry that might fit the hominin Umwelt would thus center on daylight and natural objects. The objects, recognized as such by vision, would be at least very approximately Lambertian. Indeed, anything not approximately Lambertian would not be a true visual object. In such cases, one might have a process (instead of an object), or have one *physical* object split in a number of mutually distinct *visual* objects.

*Objects* in real scenes may have a schizophrenic being, like a face seen *en face* may appear different from *the same* face seen *en profil*. The realization that *Hesperus is Phosphorus* (Frege, 1892) is of a cognitive nature. But we are discussing immediate awareness, which is precognitive. It is exactly like that with objects far from Lambertian scattering. Think of *morpho* butterfly wings. This schizophrenia may be so severe that the very notion of (visual) objecthood becomes void, as in the case of mirrors.^18^

## Biologically Constrained Colorimetry

**Figure 1.**
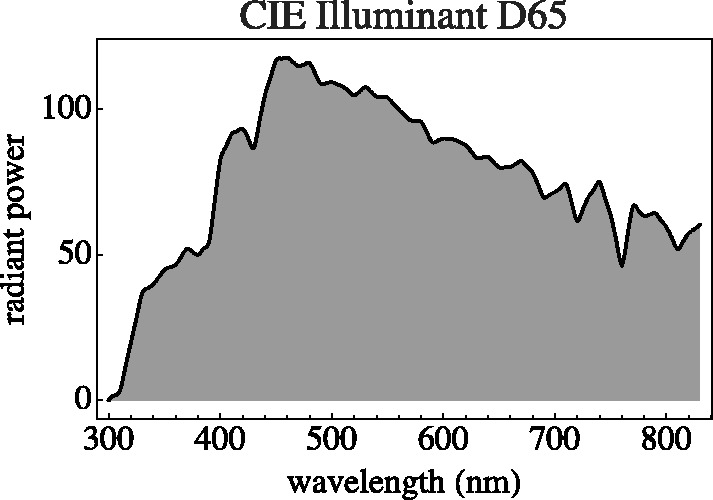
The cie d65 Average Daylight Spectrum. Here, the domain rather markedly exceeds what one might call the “visual range,” which is more like 400 to 700 nm.

A biologically constrained colorimetry focusses on roughly Lambertian objects seen under some variety of daylight. It will be convenient to select a particular standard illuminant. A convenient instance is cie illuminant d65 *average daylight* (Figure 1).

The selection of a white standard object is less arbitrary. It will have to be a perfect Lambertian surface of unit albedo. One has no choice.

To make a quick start, consider a simplified, standard setup. A number of colored papers—including the white standard—are laid out flat on a tabletop. They are illuminated with a uniform beam, say daylight from an overcast sky.^19^

The biological setting introduces ecological constraints that serve to “tame” the counterintuitive traits of pure colorimetry. For instance, it suggests that spectral articulations at arbitrarily fine scales simply will not occur and that correlations between spectral reflectance spectra and irradiance spectra have ecological probability zero. This renders our proof in the Appendix C that colors are not object properties irrelevant. In fact, colors can be shown to be excellent *distinguishing marks* for objects in the umwelt. *Roses are Red, Violets are Blue* is good enough for hominin existence.

For colorimetry to be a useful tool in the life sciences, biological relevance is a key requirement. This implies distinct colorimetries for different species. Here, we construct a biologically appropriate colorimetry for the generic human observer.

### Colorimetry in the Presence of a Standard Source

Consider a standard source. The source needs to have spectral radiant power over the full visual range. In our examples, we use cie d65 (Figure 1). The *color of the light* is apparent from the perceived color of the white object. *White light* is seen when white daisies (objects!) look white. This requires a generic context. When we refer to the spectrum of the source, we refer to the spectrum D(λ)>0 of the beam scattered by the white reference.

Arbitrary Lambertian sources cannot scatter more radiation than the white reference. Therefore, we consider radiant spectra S(λ) that are dominated by the spectrum scattered by the white reference. The beam scattered by the standard white is the envelope of all admissible radiant spectra. It dominates them. Formally, we have the constraint S(λ)≤D(λ). This is the fundamental, nonlinear, physical constraint that defines the formal structure of the realm of object colors.

Geometrically, the realm of such admissible spectra claims an infinitely dimensional *hypercrate*^20^ in the space of radiant spectra. It is convex and centrally symmetric. Because colors derive from a linear transformation (a projection) of the space of radiant beams, convexity and central symmetry are conserved (Koenderink, 2010a).

**Figure 2.**
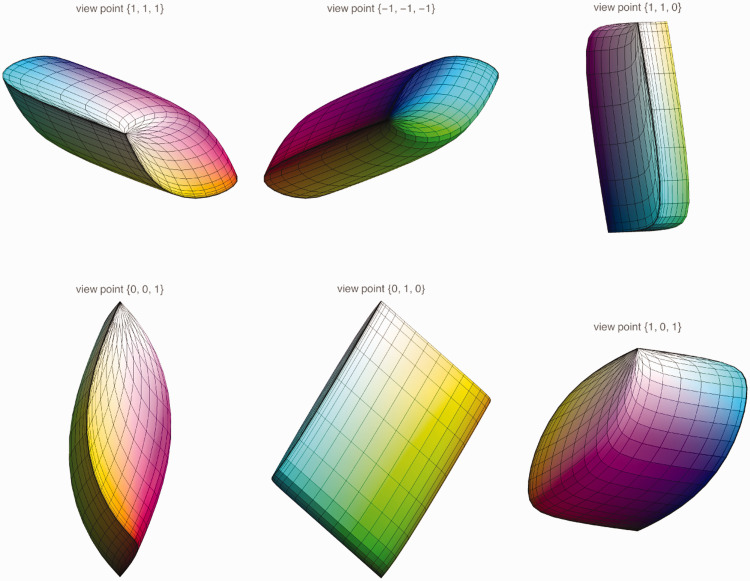
Various Perspectives on the Color Solid for Average Daylight cie d65 in cie xyz Space. (The coloring is just for convenient orientation; this is colorimetry, not phenomenology.) The color solid is a finite, convex volume in color space.

The volumetric region of colors induced by the standard source is the Schrödinger color solid (Figure 2). Schrödinger (1920) proves that the colors on the boundary of the color solid are characterized by the following:

1. The spectrum is χ(λ)D(λ) with the characteristic function χ(λ) either 0 or 1;
2. The characteristic function χ(λ) has at most two 0↔1 transitions.

This enables one to compute this object. Its surface is smooth except for singular-edge singularities and conical-point singularities (Figure 2).

In order to find a pragmatic representation, we attempt to construct the largest inscribed crate^21^ to the color solid (Koenderink, 2010a). Here, *largest* is well defined. The space of radiant spectra is only a Hausdorff space; it has no metric. But ratios of volumes are invariants of linear transformations.

**Figure 3.**
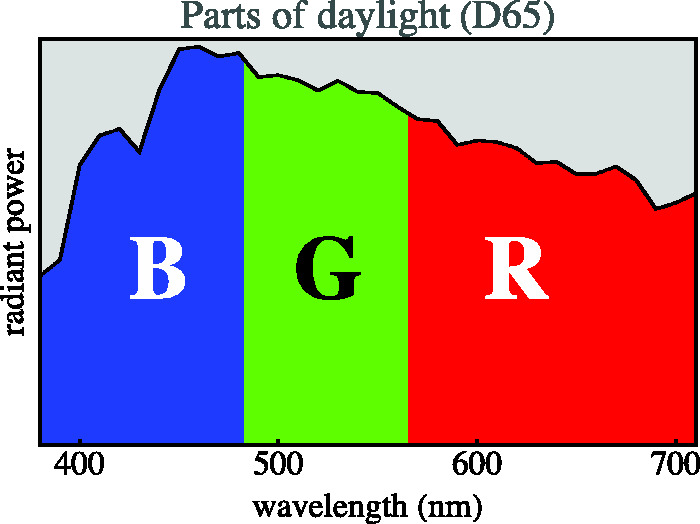
The Three *Parts of Daylight* Indicated in the Daylight Spectrum. The transitions between the parts are at 482.65 nm and 565.433 nm. An object that reflects all radiation in the blue part but nothing in the green and red parts will look blue, and so forth for green and red. The white object reflects all three parts completely. The best yellow paint will reflect the red and green parts, but not the blue. There are eight distinct such subsets; they define spectra whose colors are the vertices of the rgb cube. They are yellow, green, cyan, blue, magenta, red, white, and black.

**Figure 4.**
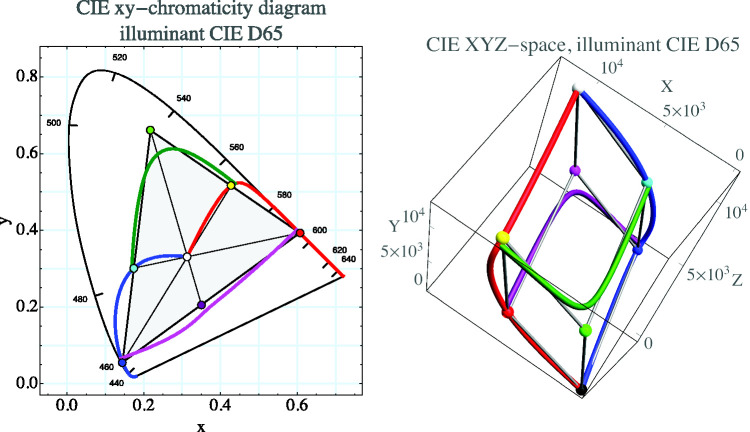
The Skeleton Structure Induced by the Color Solid in cie d65 in the cie xy-Chromaticity Diagram (Left) and in cie xyz Space (Right). The red curve is the locus of χ(λ)=1 for $\lambda >\lambda_{2}$, $\lambda_{2}\in (\lambda_{\mathrm{uv}},\lambda_{\mathrm{ir}})$, otherwise 0. The blue curve is the locus of χ(λ)=1 for $\lambda <\lambda_{1}$, $\lambda_{1}\in (\lambda_{\mathrm{uv}},\lambda_{\mathrm{ir}})$, otherwise 0. The green curve is the locus of χ(λ)=1 for $\lambda_{1}<\lambda <\lambda_{2}$, $\bar{\lambda_{\mathrm{ir}}}<\lambda_{1,2}<\bar{\lambda_{\mathrm{uv}}}$ (the bar notation $\bar{\lambda }$ indicates the complementary wavelength of *λ*), otherwise 0. The purple curve is the locus of χ(λ)=0 for $\lambda_{1}<\lambda <\lambda_{2}$, $\bar{\lambda_{\mathrm{ir}}}<\lambda_{1,2}<\bar{\lambda_{\mathrm{uv}}}$, otherwise 1. The crate has been indicated at right, its vertices colored for easy reference. At left, it appears as a triangle, as the chromaticity diagram is a projection from the origin. The triangle shows the gamut of chromaticities claimed by the crate. It is very similar to the gamut claimed by generic electronic display units. The curve marked with wavelengths is the spectrum locus, the *horse shoe shape* referred to earlier. We use this figure also to indicate that the cie xyz and rgb (remainder of the text) parameterizations can be used *ex æquo* in the representation of colorimetric structures. The rgb system yields more geometrically appealing and intuitive pictures though (e.g., Figure 5).

The crate is unique, as is found by exhaustive search. The solution is a tripartition of the visual range induced by two cut loci: $\lambda_{1}=482.65$ nm and $\lambda_{2}=565.433$ nm. Thus, the visual range is partitioned into three parts (Figure 3), namely:

**blue part** where χ(λ)=1 for $\lambda_{\text{uv}}\leq \lambda <\lambda_{1}$, otherwise 0;

**green part** where χ(λ)=1 for $\lambda_{1}\leq \lambda <\lambda_{2}$, otherwise 0;

**red part** where χ(λ)=1 for $\lambda_{2}\leq \lambda <\lambda_{\text{ir}}$, otherwise 0,

where one may take^22^ the approximate spectrum limits as $\lambda_{\text{uv}}=$ 380 nm and $\lambda_{\text{ir}}=$ 720 nm. The crate claims *ca.* 65% of the volume of the color solid (Figure 4; Bouma, 1946).

That these formally defined spectra indeed correspond to *seen* blue, green, red, and so forth is a remarkable fact of experimental phenomenology. It was noticed by Schopenhauer (1816) in an attempt to make scientific sense of Goethe’s (1810) intuitions. It is a *brute fact* because there can be no causal reason. Yet it cannot honestly be denied by generic observers.

Denoting the parts *R*, *G*, and *B*, any color (of some beam dominated by the standard) can be written as the color of *rR* + *gG* + *bB*, where r,g,b≤1. The beam *rR* + *gG* + *bB* will be referred to as the canonical representation of the color. The spectrum that gave rise to the color is metameric to the canonical representation.

The domain of the coefficients {*r*, *g*, *b*} is the unit cube $I^{3}$, which we will refer to as the “rgb cube.” The vertex {0, 0, 0} represents black (abbreviated k). The vertex {1, 1, 1} represents white (abbreviated w).

The remaining vertices may be divided into primary and secondary *cardinal colors*. The primary colors are {1, 0, 0} (r for red), {0, 1, 0} (g for green), and {0, 0, 1} (b for blue), whereas the secondary colors are {0, 1, 1} (c for cyan), {1, 0, 1} (m for magenta), and {1, 1, 0} (y for yellow). The cardinal colors occur in the natural periodic sequence ygcbmr.^23^ They are indeed “cardinal” because the only rgb cube points that lie on the boundary of the color solid. Proceeding from primary and secondary, we find that white is the unique tertiary cardinal color. Going in the reverse direction, we may add black. By construction, the pairs rc, gm, by, as well as kw are mutually supplementary (add to white). Standard colorimetry would reckon these “complementary with respect to the illuminant”; it has no natural notion of supplementarity.

**Figure 5.**
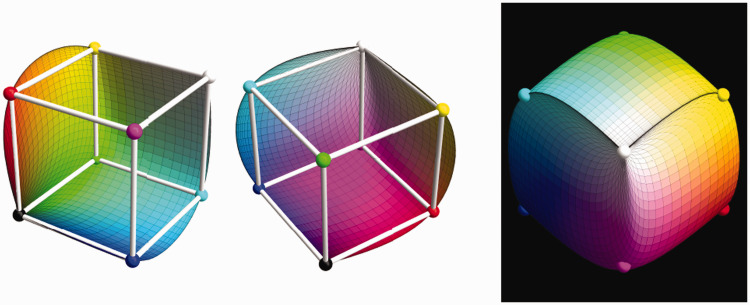
Three depictions of the color solid. At left, the rgb cube inside the color solid, depicted in rgb space. It is the largest (qua volume) inscribed crate to the color solid. Here, we split the color solid into two mutually congruent halves. The cut is by the red and blue curves shown in Figure 4 right. Notice that the color solid appears as a slightly inflated unit cube. This is also seen in the figure at right, which is a view from the outside. Notice that the vertices of the rgb cube lie on the boundary of the color solid.

All the vertices of the rgb cube lie on the surface of the color solid. The color solid (which is necessarily convex) appears as a slightly inflated unit cube (Figure 5).

On mapping databases of natural colors in color space, one finds that typically over 99% lie in the interior of the rgb cube. The ones that do not, have some coordinate only marginally different (0.01 range, see later) from 0 or 1. From a formal perspective, the rgb cube is the natural arena for the object colors (Küppers, 1978, 1989, 2005; Smith & Lyons, 1996).

**Figure 6.**
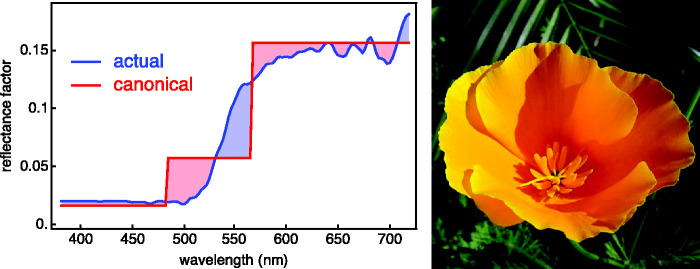
A Reflectance Spectrum of a Californian Poppy (*Eschscholzia californica* [*Papaveraceæ*]; Spectrum From the Floral Reflectance Database [FReD, reflectance.co.uk]) Flower and Its Canonical Spectrum. The poppy color under standard daylight is {0.156,0.057,0.016}, the rgb coordinates are the levels of the canonical representation.

**Figure 7.**
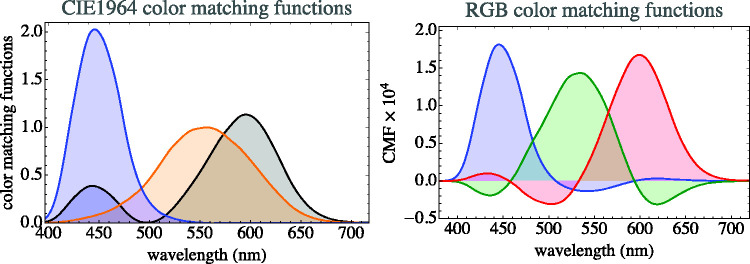
The Color Matching Functions. At left, the cie xyz color matching functions; at right, the rgb color matching functions that refer to the parts of daylight (Figure 3) as primaries. They are simply linear combinations of each other; thus, they lead to exactly the same colorimetry. (Colors used in the graphics at left arbitrarily chosen.) The rgb representation is far more intuitive in applications to the life sciences, as it allows much clearer graphical representations in virtually all cases. (In this case, the colors used in the graphics at right signify the “red” [long wavelengths], “green” [medium wavelengths], and “blue” [short wavelengths] parts of the partition of white.)

Taking the conventional display color nonlinearity (often referred to as gamma correction; Poynton, 1993)^24^ into account, the rgb cube is very similar to the RGB pixel space of jpeg images (Pennebaker & Mitchell, 2004). Thus, one has a useful map from spectra to pixel values and even from pixel values to spectra (the canonical representations). The latter observation yields a very useful heuristic with numerous applications (see Figure 6). This renders the rgb representation much more convenient and intuitive than the conventional cie xyz representation (Figure 7).

The construction of rgb space is based on average daylight. That is exactly *why* the representation is intuitive and useful in biological contexts. Without a designated illuminant, there is not much formal structure. However, because daylight may vary capriciously, one should be prepared to handle such variations (see later).

### Colorimetry of Object Colors

Again, the standard setup, that is the simplest possible setting, considers colored papers, laid out flat on a tabletop and illuminated by a uniform source, such as the northern sky, the classical illumination of artist’s studios (Friel, 2010).

Let the object gamut contain a white object. It serves to *anchor* the gamut (Gilchrist et al., 1999). This is a bare bones context from which one may deviate, or articulate into various directions.

Let the radiance of the beam scattered by the white reference be denoted D(λ). Let spectral reflectance factors be denoted $F_{i}(\lambda )$ (the index *i* ranging over the various objects). Then, the beam scattered to the eye by the ith object is $F_{i}(\lambda )D(\lambda )$. One finds the parameters $\left\{r_{i},g_{i},b_{i}\right\}$ such that $r_{i}R+g_{i}B+b_{i}B$ and $F_{i}(\lambda )D(\lambda )$ are metameric. This involves the color matching functions shown in Figure 7 (right).^25^ One also finds the parameters $\left\{r_{0},g_{0},b_{0}\right\}$ such that $r_{0}R+g_{0}B+b_{0}B$ and D(λ) are metameric. Then, one defines $\left\{r,g,b\}=\{\frac{r_{i}}{r_{0}},\frac{g_{i}}{g_{0}},\frac{b_{i}}{b_{0}}\right\}$ as the colorimetric coordinates of the *object color* defined by the reflectance *F_i_*. The division implements “color constancy” (Olkkonen et al., 2009; von Kries, 1905).

This forces the object colors to lie in the unit cube $I^{3}$, henceforth referred to as the rgb cube.^26^ The division by the white reference implements an adaptation, or anchoring, that is familiar from phenomenology (Gilchrist et al., 1999).

The white object serves to scale for the overall radiance. That is to say the illuminant D′=αD with α>0 will yield results that do not depend on *α*. Thus, we have modeled the “adaptation” that is well known to apply to a huge range of radiances in the case of human vision.

An extrapolation of this is to admit spectral variations and uses illuminants $\bar{D}(\lambda )$ instead of D(λ), while using the same formalism throughout. For the white object, this has the effect that it is invariant against spectral changes of the illuminant. It is trivially true for the black object too and thus for the kw body diagonal of the cube. It is not necessarily true for the other colors.

How useful this is, is a matter of empirical investigation. The intuition is that it should work fine for relatively minor variation about the standard illuminant. This implements the *automatic white balance* of human vision. The mechanism is similar to the “von Kries coefficients Law”^27^ (von Kries, 1905).

When the spectrum of the illuminant is changed, the color solid itself changes, and the inscribed crate can no longer be the largest one. It will remain very close to that for relatively minor spectral variations, though. Colors might conceivably move in or out of the rgb cube. In the (ecologically valid) settings considered here, this is exceedingly rare (see later).

How descriptive this model is can only be judged on the basis of variations that remain in the ecologically valid range. Thus, the issue has to be investigated on the basis of statistical models of the generic environment (Koenderink, 2018b).

There can hardly be any doubt that visual systems evolve according to *Umwelt* (both ecology and anatomical/physiological apparatus) and *Lebenswelt* (or lifestyle)^28^ (see von Uexküll, 1926, 2010).

## Ecological Variations

Colorimetry as such has nothing to say about the structure of color space, except that it is a linear space. Color space is just a projection of the space of spectra.

There are two, categorically distinct, ways to explore the metrical structure of color space. One is *psychophysics*. For instance, one might measure hue discrimination (Farnsworth, 1943; van Esch et al., 1984; Wright & Pitt, 1934), or even general just noticeable differences all over color space (MacAdam, 1942, 1947; Noorlander & Koenderink, 1983). It defines a graininess and a Riemann metric. One finds that human observers may differentiate as many as 10^6^ to 10^7^ colors (Wyszecki & Stiles, 1967). This suggests an uncertainty of about 0.01 in the *rgb* coordinate values (Koenderink et al., 2018).^29^ This is not unreasonable in view of the thumb rule of 1% for the Weber fraction of radiant powers.

Other ways to explore structure is through *phenomenology* (for instance, Munsell, 1905, 1912) or through *ecology* (Koenderink, 2018b). The latter is the main topic of this section. The biological perspective suggests that the phenomenological resolution will have evolved to match the uncertainties due to ecologically valid variations.

### The Realm of Illuminants

For diurnal, terrestrial animals, the illuminants are varieties of daylight. Daylight changes with time of day, meteorological conditions, and ambient scattering (Koschmieder, 1925; Schuster, 1905; Strutt, 1899).

Exceptions that we ignore are such radiant sources as the bioluminescence of glowworms, fire light, and so forth. Glowworm light is a vital issue in the glowworm world, even if it is a mere curiosity in ours. Different animals, different realities. *Hominins* are no less special cases than the *Lampyridæ* are. In biology (psychology is different by design), anthropocentrism implies dubious science.

There are two aspects to this: the Umwelt and the lifestyle. The Umwelt contains all the ways the environment can work on the animal. For humans, this includes radiation in the visual band but excludes radio waves. It also includes all the ways the animal can work on the world. For humans, this includes moving stones but excludes generating strong electric fields without assisting technology.

Animals may live in very different Umwelts. The lifestyle involves the use of Umwelt elements in the course of generic actions (von Uexküll, 2010). For instance, the tiger and the lamb have similar Umwelts but very different lifestyles.

The ecologically valid realm of illuminants is evidently the range of daylight spectra. Hominins might have known fire light as a case of secondary importance. Yet it is the daylight—hunting–gathering time—that would have driven their evolution.

Fortunately, there exist databases that cover daylights under parametric variation of:

**Table:** 

time of day	dusk to dawn
meteorological circumstances	open weather, overcast, fog, hydrometeors
geographical latitude	the height of sun at noon
season	the height of sun at noon, atmospheric haze
ambient scattering	scattering from vegetation, bushes, trees

and a few more. Because it is awkward to mix databases, we use just a single database from the Kohonen laboratory (Kohonen et al., 2006; examples in Figure 8). It should cover most ecological variation. This database contains 52 radiant spectra.

**Figure 8.**
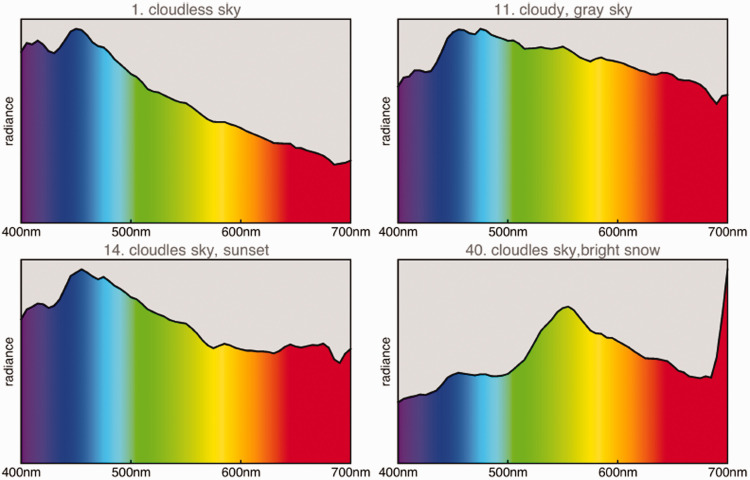
Some Instances Taken From the Kohonen Daylights Database (Kohonen et al., 2006).

This database may well be slanted toward extreme cases. Anyway, the major influence of the illuminant is by way of spectral slope (color temperature) and (already much less so) spectral curvature. The more or less random component due to environmental factors (Morimoto et al., 2019) hardly plays a quantitatively important role.

### The Realm of Objects

The primordial object for the terrestrial, diurnal animal is a volumetric region (its interior not optically revealed) bounded by an opaque, approximately Lambertian scattering surface. Think of a potato. Local surface elements of such objects scatter the same radiant beam to the eye from wherever the animal might be with respect to the object. The large majority of biologically important objects in nature are of this generic type. These include stones, earth, vegetation, animals, and processed things such as roots, wood, bones, meat, fat, and more. Even huge areas in the visual field such as the sky or large expanses of water fall in a different class. Their colors tend to be of little interest when you are hunting, or gathering, for your daily meal.

Common exceptions from our contemporary Umwelt tend to be man-made; think of polished metals. They came into existence after the evolution of hominin vision was already well on the way to its current state. A mirror *has no color*. The beam scattered to your eye depends critically on your position. In a hall of mirrors you are lost, albeit differently from being lost in the woods. In a hall of mirrors you may run into the walls, but in a forest, there is no reason to run into tree trunks. Tree trunks are bona fide visual objects, whereas mirrors are not.

It is difficult—indeed, probably impossible—to define an ecologically valid, statistically balanced, generic range of objects. It should certainly include:

**Table:** 

vegetation	leaves, flowers, bark, moss, grass
internal or subterranean parts	fruits, nuts, roots
animal coverings	skin, fur, scales, feathers
animal internal parts	flesh, fat, bones, gut
minerals	earth, stones, mineral pigments

A study of spectral reflectance factors suggests that the spectral articulations found in these classes are statistically very similar.

**Figure 9.**
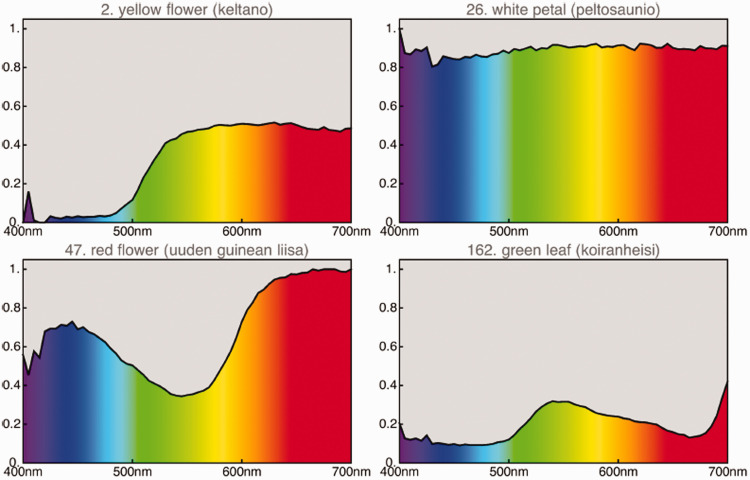
Some Samples From the Finnish Vegetation Database (Kohonen et al., 2006).

For a start, we select two databases: one on vegetation (Kohonen et al., 2006; examples in Figure 9) and one on mineral pigments.^30,31^ The vegetation database has 219 items: leaves, stems, flowers, and dry brown leaves. The mineral pigments database has 723 items of mainly classical painter’s pigments in powdered, dry form. After deleting samples with a significant specular component, 651 items are left. We let these represent the “earth colors” as different from the vegetation colors. Perhaps unfortunately, we are missing animal colors. There appears to be no suitable databases in the public domain. A spot check on spectral reflectances of bone, fat, meat, and skin suggests that it would hardly change the picture much, if at all. We feel that we cover the realm sufficiently.

Perhaps regrettably, we have no data on the frequency of occurrence or on the relative relevancy for the hunter-gatherer lifestyle. Our choice is likely to overrepresent singular outliers. But perhaps this is not such a great disadvantage. We are likely to obtain a somewhat pessimistic view of the ecological graininess.

**Figure 10.**
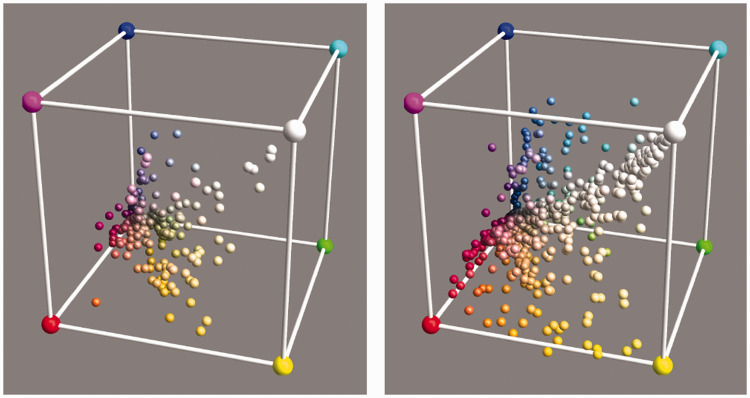
The Distributions of the Vegetation Database (Left) and the Mineral Pigments Database (Right). These are the colors under the standard source cie d65.

Neither database fills the rgb cube more or less homogeneously (Figure 10). This is indeed typical of all ecologically valid databases. The convex hull of the colors in the vegetation database claims 21% of the volume of the cube and that of the mineral pigments claims 44%. Because convex hull volume is essentially determined by the outliers, these are certainly overestimations.

Figure 10 yields a good illustration of our earlier remark that in practice almost all object colors lie in the interior of the rgb cube. For the vegetation database, there are three cases (out of 219) in which the green coordinate is less than −0,01, the actual values being –0.0186, –0.0171, and –0.0118; there are no values greater than 1.01. For the mineral pigments (among more containing the most vivid artist’s pigments), there are 5 cases in the red, 35 in the green, and 3 in the blue with coordinate values less than 0, and there are 2 cases in the red for which the coordinate values exceeded 1. In the former case, the median undershoot is 0.018, and in the latter, the overshoot is 1.026. This is typical; it is one reason why the rgb representation is so useful.^32^

### The Graininess Induced by Illumination Changes

**Figure 11.**
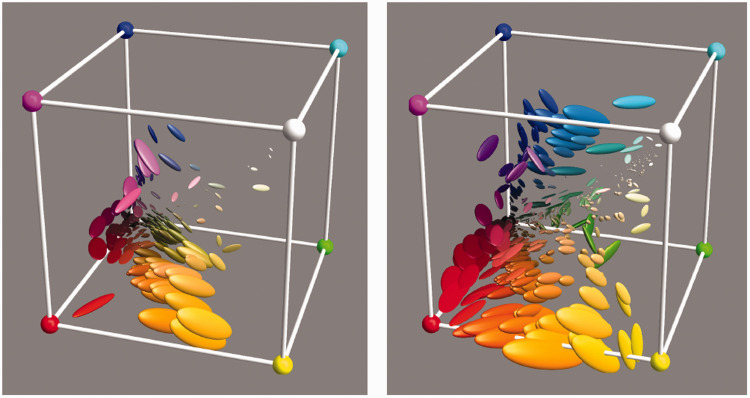
The 95% Confusion Ellipsoids Due To the Variation of the Illuminant for the Items in the Vegetation Database (Left) and the Mineral Pigments Database (Right).

A very straightforward investigation simply puts all objects under all illuminants. Thus, we obtain 52 colors for each object. To streamline the presentation, we summarize these sets by covariance ellipsoids at the 95% level (Figure 11).

We find that the automatic white balance works really well. This perhaps reminds one of results obtained by hyperspectral imaging (Nascimento et al., 2002). Although the illuminants vary over the full daylights database, the various items stick quite close to the location they have when illuminated by the standard source (Figure 10).

The capricious variations of color appearances, noticeable as deviations from *color constancy*, cannot be accounted for by the observer on the basis of available visual observations. They are due to *hidden variables* and thus naturally occur *magically*.

We define the *equivalent radius* of an ellipsoid as the radius of the sphere with the same volume as that ellipsoid. We also define the *maximum radius* as the maximum semiaxis of the ellipsoid.

For the vegetation database, the range for the equivalent radius is (0.000,0.059), and the quartiles are (0.006,0.013,0.02). The range for the maximum radius is (0.000,0.165), and the quartiles are (0.019,0.034,0.058).

These numbers are in terms of the edge length of the rgb cube; thus, they are quite small. The mean ellipsoid volume is only $5.4\;10^{-5}$ of the volume of the rgb cube. This implies a Weber fraction per rgb coordinate of a few percent (take the cube root); thus, it exceeds the psychophysical discrimination level. It provides for about 20,000 (take the reciprocal) distinct colors, much higher than numbers from experimental phenomenology (about a thousand).

For the mineral pigments database, the range for the equivalent radius is (0.000,0.076), and the quartiles are (0.002,0.008,0.019). The range for the maximum radius is (0.000,0.0076), and the quartiles are (0.006,0.024,0.053). Thus, there is not much difference in the results for the two, ontologically very different, databases.

The confusion ellipsoids are evidently not isotropic. There is apparently an overall trend, although there is hardly a smooth field on the local scale. One reason for a trend is probably the fact that the daylight spectra differ mainly in one, at most two dimensions (the spectral slope and curvature, the slope being the dominant factor). The more or less abrupt variations are expected. They are doubtless due to the idiosyncrasies of the object spectra. These differ from the corresponding canonical spectra in manifold ways. One expects that taking the metamers of the object spectra into account would tend to smooth things out.

### The Additional Graininess Induced by Metamerism

Both conceptually and technically, metamerism poses a problem. It is quite clear what has to be done in principle though, namely:

1. Given a color, determine the ecologically valid set of (mutually metameric) spectra that will yield this color under the standard illuminant;
2. Determine the color of each of the metamers as seen under all ecologically valid illuminants;
3. Summarize the resulting distribution, for instance, as a covariance ellipsoid;
4. Repeat this for all colors in the rgb cube.

Problems are that the color is not likely to be in the database and that the database will not yield a set of representative metameric instances. For investigations of this type, the existing databases are hugely insufficient. The only way out is to summarize the databases in terms of generative statistical models (Griffin, 2019; Koenderink, 2010b; Koenderink & van Doorn, 2017; Zhang et al., 2016).

Of course, setting up such statistical models is a worthwhile scientific enterprise by itself. It has to be one of the fundaments required in studies of the evolution of the human visual system.

To be able to proceed, we first need to consider some basic complications one immediately runs into in an attempt to implement this program. These mainly involve methods to deal with the nature of the domains. Such methods should not rely solely on formal methods. They need to be rooted in concepts from basic physics.

#### Dealing With the Nonlinear Constraints

The computational machinery of formal colorimetry is linear algebra. This cheerfully operates with entities that have no possible physical existence. Such entities (such as the elements from the null-space of the color matching matrix) may well represent very useful virtual objects. However, eventually both the input and the output of computations need to represent *physical* entities.

Physics introduces a number of constraints on the objects that formal colorimetry works with. In a more positive vein, the physical constraints yield very important structures. The color solid itself is a prime example of that.

Failure to reckon with physical constraints easily leads to erroneous results. A common instance involves nonphysical predictions being “cured” by clipping. Principal components analysis (PCA) is often made to work that way.

It should be recognized when something is fundamentally flawed. Correct methods should make it impossible to *ever* obtain nonsensical results. In most cases, the solution is to use some variety of homomorphic filtering (Oppenheim et al., 1968).

#### The Nonnegativity of Radiant Power

Radiant power is necessarily nonnegative. An obvious way to deal with this is to consider the logarithm of radiant power (Koenderink & van Doorn, 2017). This is very natural from the perspective of physics. Most interactions involving radiant power are of a multiplicative nature. Another reason is that the fully noninformative Bayesian prior is hyperbolic, therefore uniform on the log scale (Jaynes, 1968, 1973). The general method to deal with the constraint is to use a variety of homomorphic filtering. One maps the radiant power to the logarithmic (one might call it the *physical*) domain, operates on it (e.g., does various statistics), and transforms back.

In the physical domain, one may use linear methods, as the structure of ${\mathbb{R}}^{+}$ was transformed into A, the full affine line. This solves any sign problems with PCA. Another problem with PCA is that it requires a *metric*. In using PCA, a default metric tends to be silently (or even upfront; Lenz, 2002) assumed. It really should be a first consideration. It requires reasons based on considerations of physics. Here, the logarithmic transform also helps out, because the Bayesian prior becomes uniform.

#### Dealing With the Finite (0,1)–Reflectance Range

The domain I, the unit interval, also renders the use of linear methods, such as PCA, fundamentally flawed. Nevertheless, it is often used (Chiao et al., 2000; Lenz, 2002; Maloney, 1986). Only sheer luck prevents sign, or overflow problems to emerge. The conventional solution to simply clip results to the unit interval is a mere kludge.

The reflectance is ultimately due to optical processes that depend on physical parameters such as absorption and scattering cross sections. Such physical quantities are defined on the nonnegative reals, suggesting that one should identify the appropriate physical domain. This is complicated, because various processes may account for ecologically relevant reflectance factors.

Perhaps the dominant process is well described in terms of the Kubelka–Munk theory (Joshi et al., 2001; Kubelka, 1948, 1954; Kubelka & Munk, 1931) of radiative transfer in layered, turbid media. This applies to papers, paints, and textiles in our present society (Hubbe et al., 2008; Judd & Wyszecki, 1975; Pauler, 2001; Stenius, 1951a, 1951b, 1953; Van den Akker, 1949). Perhaps more appropriately, it equally applies to earth, vegetation, skin, and bones in the hunter-gatherer’s world.

The reflectance depends upon the ratio of the absorption *K* and scattering *S* cross sections. This *spectral signature K*/*S* is widely used in materials research. It only depends on physical quantities that live in ${\mathbb{R}}^{+}$. This suggest that the appropriate physical domain is the logarithm of the spectral signature, that is log⁡(K/S).

**Figure 12.**
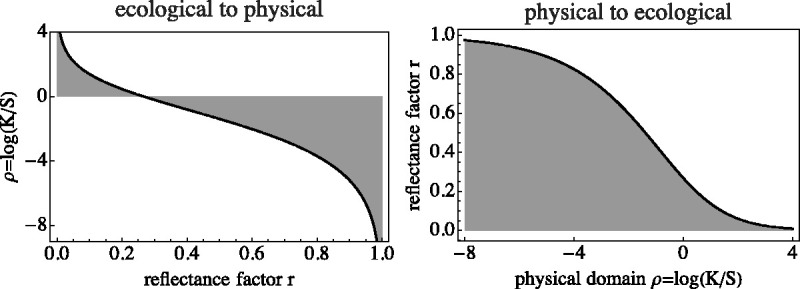
The Function *f*(*r*) (Left) and g(ρ) (Right). Notice that f:I↦ℝ, whereas g:ℝ↦I.

The spectral signature depends only upon the scattering and absorption cross sections and thus is a true *object property*. Using the Kubelka–Munk theory, one can move back and forth between the spectral reflectance and the spectral signature. To do that, we define a pair of mutually inverse functions f(r)) and g(ρ) (Appendix A, Figure 12).

The spectral signature is simply related to the reflectance factor of an optically thick turbid layer. The function f(r)) maps the unit interval I on the full affine line A, and the inverse function g(ρ) maps the full line to the interval (Figure 12).

That this is generally a good idea is clear when one compares histograms of pixel values in photographs of vegetation (tundra or savannah-like scenes). Although the raw pixel histograms tend to be complicated, often bimodal, the histograms in the physical domain tend to look much more unimodal and symmetric (Koenderink & van Doorn, 2017). It is apparently the more appropriate domain for statistics. This is another instance of homomorphic filtering. In any case, one tries to map from the ecological to some appropriate physical domain.

One may meet with a variety of physical processes. In any specific case, one identifies the most appropriate homomorphic representation. This calls for a broad familiarity with classical physics (Feynman et al.,1964–1966).

#### Statistical Models of the Illuminant

For the purpose of colorimetry in the context of the hominin Umwelt, the absolute levels of radiant power are largely irrelevant. It is mainly the spectral distributions that count. So the first thing to do is to somehow normalize the individual items, perhaps by shifting them in the physical domain.

All instances are eventually due to sunlight, filtered by the atmosphere and affected by ambient scattered radiation. These are all multiplicative processes that become additive in the physical domain.

Sunlight derives from the sun’s photosphere and is essentially thermal radiation of a $5777^{{}^{\circ}}\text{K}$ black body, articulated by a number of atomic absorption lines. Filtering by the atmosphere introduces additional molecular absorption, as well as the effect of extinction due to Rayleigh scattering and perhaps ærosol scattering. This basic structure will reappear in all instances. Thus, the ensemble mean in the physical domain is meaningful.

Time of day and height above the horizon are expected to lead to changes of overall slope and perhaps curvature. These are most properly caught by the low-order PCs. Remaining variations are likely to be due to a variety of random causes. These are most appropriately modeled by some fractal signal. There is perhaps something to say for using Planck’s formula for thermal radiation augmented by a low-order polynomial model for the deterministic part. For many applications, that would avoid overfitting. In our application, the microarticulation had perhaps better be retained. They might accidentally correlate with microarticulations in spectral reflectance factors.

**Figure 13.**
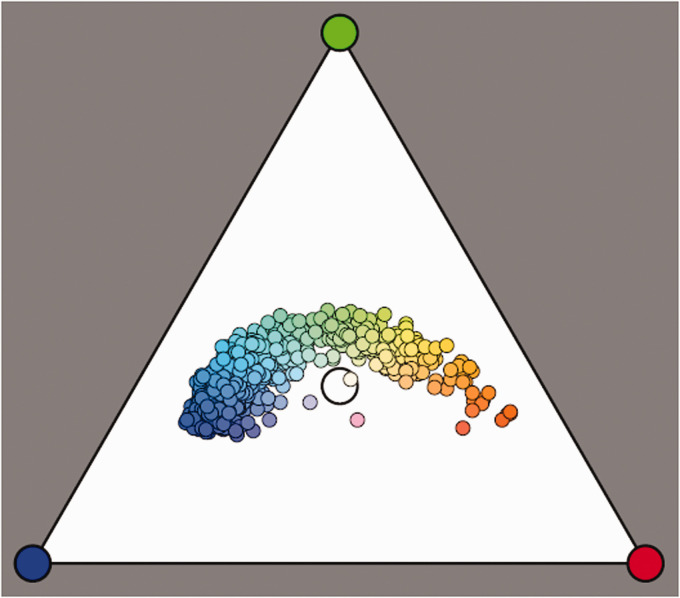
Chromaticities of a Number of Random Daylight Instances. This convenient (because symmetrical in r, g, b) chromaticity diagram is the projection from the black point on the triangle spanned by the red, green, and blue vertices of the rgb cube. It was used by Maxwell and by Helmholtz as a natural representation. The cie has pushed the deformed version (cie xy chromaticity diagram) that includes properties that not belong to colorimetry per se (luminance) and that can be found all over the Internet.

The main decision is the level at which the deterministic (PCA) low order should give way to some random fractal. It appears to be the level after the PCs that essentially represent curvature or perhaps cubical structure. This yields a simple model that may serve to generate novel instances of “daylight spectra” (Figure 13).^33,34^ We use the ensemble mean, a linear combination of the PCs with coefficients generated by a multinormal distribution (using the distribution of projections of the database instances on the PC-basis to define the covariance matrix), and a fractal fuzz. Finally, we transform to the ecological domain.

The nature of the “fractal fuzz” is estimated from the slope of the power spectrum of the residual structure, after subtracting the PCA approximation.

#### Statistical Models of the Objects

In this case, the absolute level *is* important. Thus, we do not normalize in any sense. We just move to the physical domain, assuming radiative transport in turbid layers as the dominant process. There is no a priori reason to expect a bias in spectral slope or curvature. One expects the spectral articulation to be translation invariant in a statistical sense. The reason is that the visual range is very narrow and does not involve switchovers between distinct physical processes. Molecular mechanics dominates the infrared, atomic electronic transitions the ultraviolet, whereas the visual range largely involves various changes in chemical constitution induced by the impinging photons (Feynman et al., 1964–1966). This is also the reason why this range is of major biological significance.

**Figure 14.**
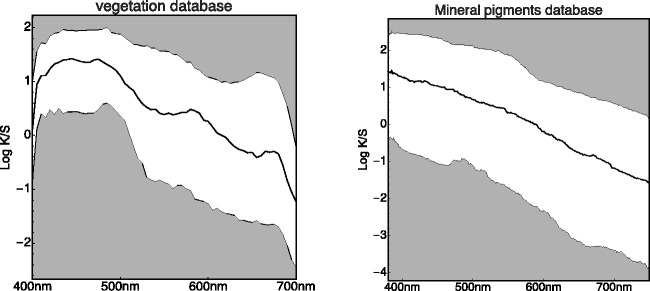
Summarized “Spectral Histograms” of the Vegetation Database (Left) and the Mineral Pigments Database (Right). “Summarizing” implies plotting the median and quartile ranges as a function of wavelength. In addition (not shown here), one tests for approximate normality of the distribution as a function of wavelength. (*Note*. The logarithm used here is the natural logarithm, different from Figure 15. Whenever the base 10 logarithm is used, we use the notation ${\;}^{10}$Log.)

To have a rough check, we compute the histograms of spectral radiant power density over the whole vegetation database as a function of wavelength (Figure 14, left). One finds some structure that might well prove specific for the vegetation database. We also find an overall slope (see also Griffin, 2019) that might well have a generic relevance.

At first blush, it perhaps looks like an imprint of the chlorophyll absorption spectrum. One way to check this is a comparison with databases of very different materials, say minerals (Figure 14, right). Here, we find the same slope, so the chlorophyll bands, typical for vegetation, are apparently not the issue. It rather is something generic that should at one point receive an explanation in terms of ecological physics. The items from the minerals database have a higher resolution (380–780 nm at 1 nm intervals); moreover. the database volume is larger than that of the vegetation data. As a result, the spectral histogram is less noisy. One notices a well-defined spectral gradient of the spectral signature with wavelength. Notice the similar trends in these very different databases. We have not seen such summaries as in Figure 14 elsewhere (but see Griffin, 2019). They evidently are a must-have in all cases.

The average wavelength dependence probably derives from the size of the microparticles in the granular media, as chemical properties are apparently irrelevant. Particle sizes are in the ten to a hundred micrometers range; thus, neither Rayleigh nor Mie scattering theories apply. The type of analysis that might apply is discussed in Pilorget et al. (2016).

The behavior near the uv spectrum limit seen in Figure 14 may be due to instrumental problems. It is absent in a huge database obtained from hyperspectral imaging,^35^ which otherwise reveals the same pattern. We have only used the latter database to check whether trends are similar to the databases used here (they are). The reason we do not use this huge hyperspectral database further is that this database seems very uneven and is likely to be troubled by systematic errors pertaining to large subsets of items.

**Figure 15.**
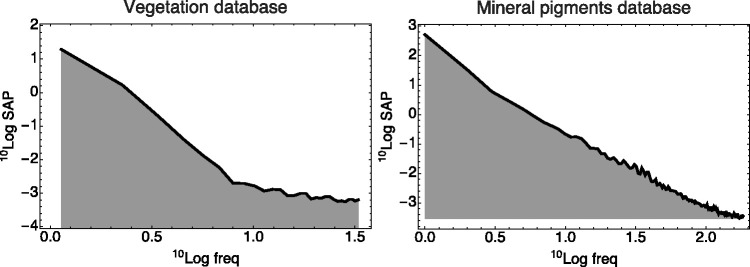
The Power Spectrum of the Spectral Articulations of the Vegetation Database (Left) and the Mineral Pigments Database (Right). The frequency is in cycles per visual range. (We let sap stand for “spectral articulation power.”) Signals were carefully windowed and so forth. The two databases are available on slightly different spectral ranges. One notices a fall off by an inverse fourth power. Such spectra are another must-have for any database.

Because the flower spectra look rather diverse, a generic model might simply be a random fractal. We find the Fourier power spectrum of the spectral articulation (of course, in the physical domain; see Figure 15). It turns out to fall off as the inverse fourth power of frequency in all databases we tested. Thus, a fractal dimension of two seems indicated. This suggests a simple model of the phenomenological optics (see also Griffin, 2019).

The spectrum will be the result of various (perhaps many) mutually independent physical causes. Each process will imprint a local structure on the spectrum at some random location. For the present purposes, the spectral width of the local structures is the relevant property. For a start, one considers just a single width and a random, uniformly distributed location. A simple, generic model of this physics is provided in Appendix B (see Koenderink, 2010b; Koenderink & van Doorn, 2017).

The model allows a formal description of the autocorrelation function of the spectral articulation. The formalism relates it to the power spectrum. From the empirical power spectrum, we estimate that the halfwidth of the autocorrelation function is about the width of the visual range (Koenderink & van Doorn, 2017). This implies that natural spectra are not highly articulated. They vary only gradually with wavelength.

**Figure 16.**
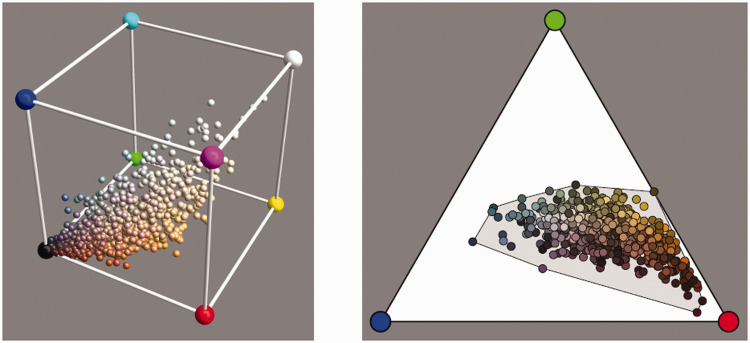
The Colors of a Number of Instances From the Reflectance Algorithm Fail to Fill the rgb Cube (Left, Samples; Right, Their Chromaticities [see Figure 13 legend] and the corresponding convex hull). This is expected (compare Figure 10).

This suggests a very simple algorithm to generate instances.^36,37^ One generates a fractal and adjusts its variance to that encountered in the database. One adds either the database mean, or just a constant slope. Converting from the physical to the ecological domain then yields the instance. As seen in Figure 16, such instances fail to fill the rgb cube. This is only natural (Koenderink, 2010b) but is also inconvenient (see later).

**Figure 17.**
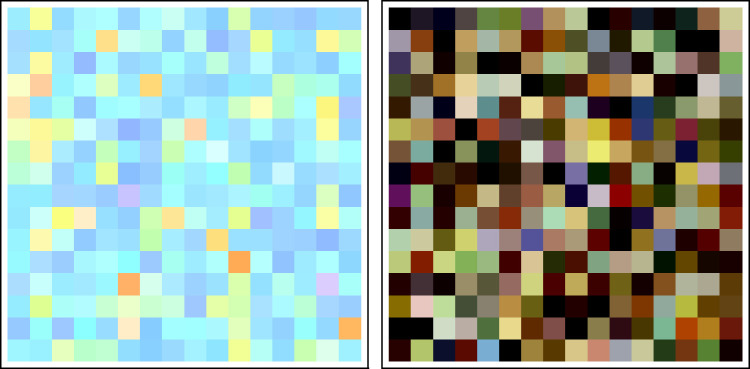
Instances of Daylight Colors (Left) and Object Colors (Right) Generated by the Statistical Models. The daylight colors have been normalized to have the maximum rgb coordinate value 1; the object colors are shown under the standard cie d65 illuminant. The models proper generate spectra, of course, not colors.

In Figure 17, we show examples (for clarity only 16 × 16 = 256 samples each) of instance of daylight and object colors generated by the statistical models.

#### Generating Metamers

For this investigation, we need to be able to generate arbitrarily many metamers for any given color. This is substantially complicated by the constraint that the metamers should be ecologically valid. Generically, there exist infinitely many mutually metameric spectral reflectance factors that will generate the given color under the standard illuminant. However, the overwhelming majority fails to be acceptable as an ecological valid reflectance. Thus, one needs to design specific methods for this case.

The databases let one generate ecologically valid metamers as linear combinations of random instances. Given three of such instances, one may combine them to yield the given color. The result will be reasonable if the three instances have colors close to that of the given color. Here starts a problem; for the databases, let one find colors only in a part of the rgb cube. Moreover, the databases yield not sufficient variation in the neighborhood of any item.

One (partial) solution is to limit the investigation to colors within the convex hull of the database colors. From a pragmatic perspective, this is not even such a bad idea. Colors far outside the convex hull are likely to be irrelevant anyway.

For more formal investigations, a way out of this problem is to generate metamers as metameric variations of canonical spectra. Because there always exists a canonical instance, this is guaranteed to work for all colors. All one needs to do to generate metameric variations is to add random metameric blacks to canonical spectra. Of course, such metameric blacks should themselves be ecologically valid.

**Figure 18.**
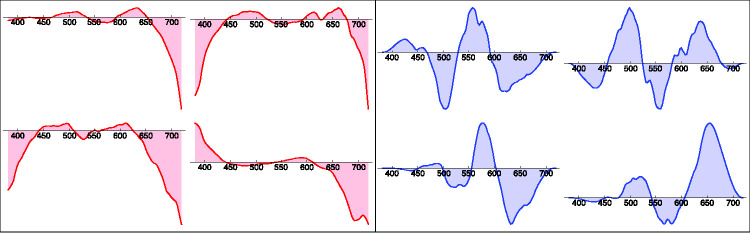
Four Random Black Reflectances Without (Left) and Four With (Right) the Use of a Hanning Window. In practice, such apodization is necessary.

**Figure 19.**
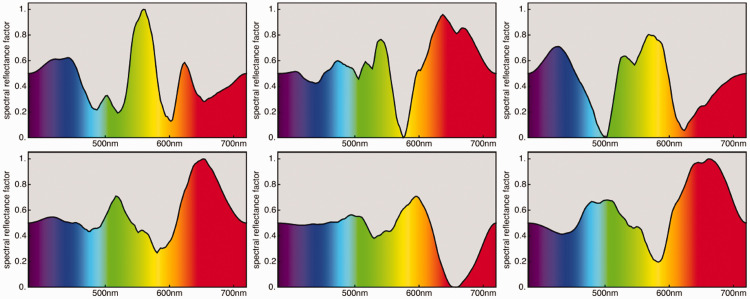
These Are Metameric Spectral Reflectance Factors for the Average Gray Color {0.5,0.5,0.5}. The black reflectances have been maximally scaled, that is, till the reflectance values hit one of the zero or one constraints. These are obviously quite “bad” grays, although they—by design—appear perfectly identical under the standard illuminant.

One easily generates such metameric blacks by generating four random instances and combining them linearly so as to obtain a black color modulo the amplitude.^38^ Unfortunately, this tends to lead to unreasonably large amplitudes near the spectrum limits (Figure 18).^39^ It renders the metameric blacks ineffective due to the physical constraints. Because the reflectance should be in (0, 1), the maximum amplitude of a perturbation is limited by that (Figure 19). A simple cure is to use a Hanning window^40^ over each of the four instances. This leads to metameric black spectra that have appreciable amplitude in the region that counts and are statistically much closer to the databases (Figures 18 and 19).

We construct random metameric reflectance spectra for a given color by adding ecologically valid black spectra to the canonical spectrum. This allows us to reach any point in the rgb cube. Unfortunately, this does not take account of the probability density of metameric blacks. This no doubt depends upon the location in the rgb cube.

It is a priori clear that colors on the boundary of the color solid are unique, that is, have no metamers. This is because the corresponding spectra have values of either 0 or 1, thus cannot be freely perturbed. For our choice of canonical spectra, this also applies to the boundary of the rgb cube.

The probability density of the metameric black amplitudes is something we do not have access to. We simply use the maximum values; thus, our estimates of the influence of metamerism are certainly overly pessimistic. The examples (Figure 19) of metameric grays reflect that.

#### The Overall Graininess Due To Ecological Variation and Constraint

**Figure 20.**
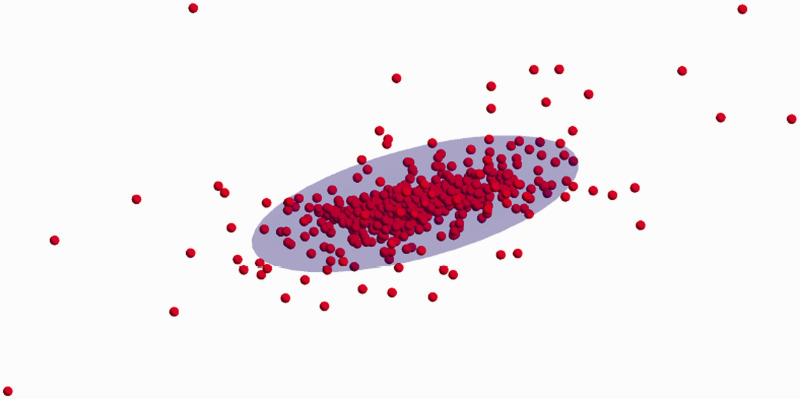
A Thousand Samples at Median Gray (Red Points). The bluish ellipsoid is the 95% boundary. This yields a good notion of the nature of the summary description by way of ellipsoidal volumes (bluish volume).

In an extensive investigation, we start with any color coordinates, call it the fiducial color. We find its canonical reflectance and perturb it with a random black. Next, we find a random instance of a daylight spectrum. Then, we compute the color and the color of the white reference, and from these, the new color coordinates. These latter will typically differ from the color coordinates we started with. We repeat this a hundred or a thousand times (say) and determine the covariance ellipsoid of the resulting set of colors that surround the fiducial one.^41^ In Figure 20, we show an example, a thousand instances at the center of the rgb cube, {0.5,0.5,0.5}.

**Figure 21.**
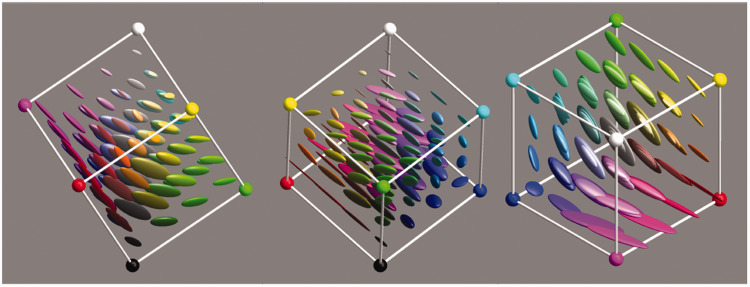
The 95% Confusion Ellipsoids in rgb Display Space (γ=2.2). This yields a realistic view on the size of the ellipsoids in jpeg photographs with automatic white balance. At left, a view that shows the black–white and green–magenta dimensions to best advantage; at center, a view that shows the black–white and red–blue dimensions to best advantage; and at right, a view that clearly shows the distribution over hues.

Repeating this procedure for many colors, we obtain a sampling of the field of covariance ellipsoids over color space (Figure 21). This defines the grain due to metamerism for the given ecological constraints. As expected, the confusion regions are larger than in the case of mere illuminance variation (Figure 11). This is due to the inclusion of metameric reflectances. The range of the maximum radius is (0.038,0.220), and the quartiles are 0.087, 0.119, and 0.145. The range of the equivalent radius is (0.012,0.079), and the quartiles are 0.033, 0.041, and 0.051.

The mean ellipsoid volume is $4.17\;10^{-4}$; thus, one estimates that there is ample room for over a thousand distinguishing marks. Thus, the proof (see Appendix C) that colors are not fit as object properties (in general!) is robbed of much of its grim impact. The proof simply does not apply to niches, like the human hunter-gatherer tundra or savannah existence. In retrospect, this was to be expected. The present calculations pin it down quantitatively.

The mean ellipsoid volume of $4.17\;10^{-4}$ suggests a Weber fraction per rgb coordinate of about 7% to 8%. This far exceeds the psychophysical discrimination thresholds but is comparable to estimates of color reproduction from experimental phenomenology (Koenderink et al., 2016, 2018). These estimates are roughly comparable with estimates based on hyperspectral images of natural scenes (Linhares et al., 2008; Marin-Franch & Foster, 2010).

## Discussion

### The Graininess of Human Color Vision

Psychophysical grain size data show that humans may resolve in the order of 10^6^ to 10^7^ colors (Pointer & Attridge, 1998). Such psychophysical data are based on just noticeable differences (jnds) that consider only the *segmentation* function. They disregard the function of color as *label*, or as distinguishing mark. The jnds are likely due to physical (photon noise) and physiological factors. A number like 10^6^ is at least three orders of magnitude larger than the grain size suggested from our investigation of the ecology.

In a recent investigation (Koenderink et al., 2018), we had 50 observers synthesize a total of 3,000 randomly selected colors using a color picker (Koenderink et al., 2016). The task made it impossible to rely on a common boundary, or transition region. The observers had to look back and forth between the fiducial and the target that they had to adjust. It was also impossible to compare either fiducial or target to a common background. In such a setting, the errors become *very much larger*. We found that humans resolve about 10^3^ rgb colors if they are forced to use colors as distinguishing marks. Physical or physiological factors cannot be responsible for this grain size. These data address the factors of biological relevance (Gegenfurtner & Rieger, 2000; Koenderink, 2018b; Olkkonen et al., 2009) as opposed to physical limits.

The precise distribution of the covariance ellipsoids found in our study (Koenderink et al., 2018) is not clearly reflected in the calculations (Figure 21). This is perhaps to be expected, as the calculations take no account of ecological or ethological weights. However, the overall magnitudes are in the right ballpark. The difference of three to four decades in size between these ellipsoids and the jnd volumes may appear huge. However, taking trichromacy into account, it drops to a factor of perhaps a little over 10 (take the cube root). This renders the number intuitively acceptable.

The grain induced by ecological variations limits the number of colors that might be used as distinguishing marks under all circumstances to about 10^3^. This seems amply sufficient for most hunter-gatherer needs. Colors as distinguishing marks are really useful in the *ecological setting*, although they are formally proven useless *in general*.

### Colors as Object Properties

*Are colors object properties?* Our findings immediately relate to this question. It addresses an issue that has been widely debated in philosophy (Byrne & Hilbert, 2003). From a general and logical perspective, the answer has to be in the *negative*. Our formal, constructive proof in the Appendix leaves no doubt. It degrades colors to mere mental paint. Neither are Roses Red, nor are Violets Blue.

However, colors remain useful labels of things from the ecological perspective. Thus, the biological answer to the question has to be in the *affirmative*. For all practical purposes, colors *are* object properties. This conviction is reflected in the significance conventionally attributed to the colors of national flags. Roses are Red, Violets are Blue. The question of whether colors are object properties is an academic one. “Object properties” or not, colors are certainly useful as distinguishing marks. They boost biological fitness.

This will be hardly surprising from most people’s intuition. Color is obviously one distinguishing mark that helps deciding between tomatoes and lemons. Perhaps oddly, a scientific backing of this intuition is lacking thus far. Our study solves this issue from a pragmatic, biological point of view—at the same time putting the intuitions of philosophers in the right. Colors are useful distinguishing marks for a hunter-gather existence in tundra or savannah ecologies.

Note that this does in no way imply that colors may not have other biologically important uses. For instance, they are instrumental in the presemantic segmentation of the optic array. For such uses, it is only relevant to be able to mutually distinguish colors, not to be able to identify, or recognize any of them. However, color used this way is not categorical, thus has no relation to colors as *qualia*. Having spectral discrimination is by no means the same as having “color vision.”

Of course, colors may occasionally fail as distinguishing marks in artificial environments. Las Vegas casinos are a case in point. This is even more so as radiant sources based on rare earths electronic structures become increasingly cheap and widely available.

The philosophical issue is by no means decided by this. Any “solution” will have to shift the formal definition of what counts as an “object property.” From a biological perspective object, properties are distinguishing marks for “things,” affordances that play a role in the agent’s life world.

No distinguishing mark is expected to be decisive in isolation, except—perhaps—in cases of the ethological releasers. An example is the impact of the red of the male stickleback belly for the female stickleback (Tinbergen, 1963). Typically, biological agents use as many distinguishing marks as are available over their various sense modalities. A few tend to suffice to clinch the matter, as smallish, independent^42^ probabilities combine multiplicatively. But even a single distinguishing mark may elicit a (possibly vague) anticipation. In biology, “object properties” can be *more or less*; they are not like the philosopher’s *all or none*. Nor deals biology with absolute truth. Evolution is driven by statistics, not (classical) logical values.

How many distinguishing marks does one need anyway? If two berries have the same color, you may always consider the habitus of the branch from which they hang. Distinguishing marks are usually used in connection with one or two (independent!) others. Coincidences of relative rare marks soon amount to practical certainty; for small probabilities of mutually uncorrelated instances, multiply.

The metameric variations of “the” color of an object do not find a possible explanation in the agent’s Umwelt. They are due to *hidden variables* that are the spectral articulations not resolved by the color matching functions. In the absence of God’s Eye’s view (the position of a biological organism), there is no way to reckon with that. The reason is the nonlinear (multiplicative) relation due to surface scattering. This magically promotes two mutually distinct sets of hidden variables into the observable domain.

There evidently is something fishy about the general, “perfectly conclusive” proof. The reason is twofold. Both source and reflectance spectra are of *limited articulation*. The spectral correlation is appreciable over the visual range. Even more important, source and reflectance spectra are essentially *never mutually correlated*. This is due to their distinct physical origin. Thus, the major premisses that enable the proof in the Appendix C are rendered ineffective due to ecological factors. The *magical* effects of the hidden variables are (fortunately!) only minor.

In the final instance, this is why human color vision *works*.

### Outlook to Further Progress

We have outlined a set of methods and a framework that enable one to study the influence of ecological factors on the utility of colors as distinguishing marks of significant objects in the hunter-gatherer tundra or savannah environment. That is the context in which the hominin visual system developed. The methods are fully general and can immediately be applied to any species, not just mammals.

Such methods allow a perspective that has been sadly underrepresented in the literature of (human) color science. For instance, the fact that human observers are able to discriminate over a million colors is enthusiastically cited, but it gains a novel (in-)significance in view of data such as presented in Figures 11 and 21.

The applicability of these methods depends critically upon the availability of relevant databases. Unfortunately, there is not much to choose from, and novel data are not readily forthcoming. One wishes that hyperspectral imaging techniques will change that. This would also yield a possible handle on the ecological/ethological weights.^43^ This is a topic that we unfortunately were not able to address.

If the desired data are not forthcoming soon (which appears likely), the only way to progress appears to be the development of generic models of source and reflectance spectra. This appears a definite possibility in view of such findings as shown in Figures 14 and 15. Such models will have to depend upon very generic properties of the physics of the terrestrial environment.

## Figures and Tables

**Figure B1.**
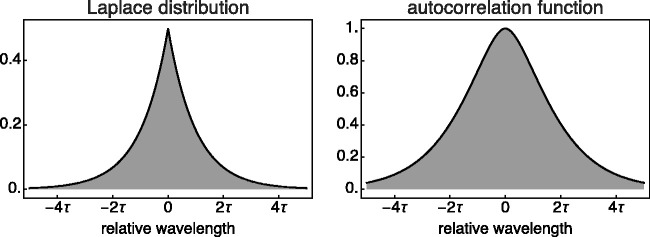
The Laplace Distribution (Left) and the Autocorrelation Function (Right). A reasonable estimate of the parameter *τ* is about 100 nm

**Figure C1.**
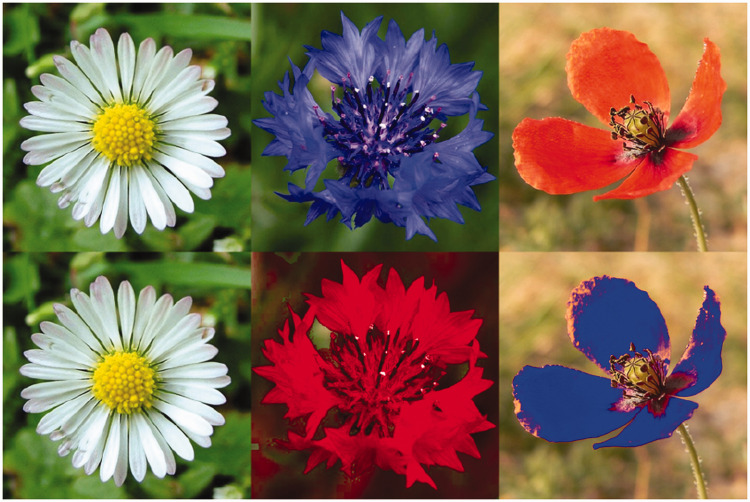
Example of three flowers. Suppose you saw the three flowers in the top row and after blinking an eye saw what is depicted in the bottom row. What happened? The white daisy did not change; thus, the (“white”) illumination apparently did not. Yet the poppy and cornflower interchanged their red and blue hues. Magic? Whatever happened, it must be the case that the red hue is not a poppy property nor is the blue hue a cornflower property. Is this experience possible at all? Perhaps remarkably, yes, it *is* possible. At least in principle. (In this case, the lower row was obviously “Photoshopped,” so these photos are not an empirical proof!)

**Figure C2.**
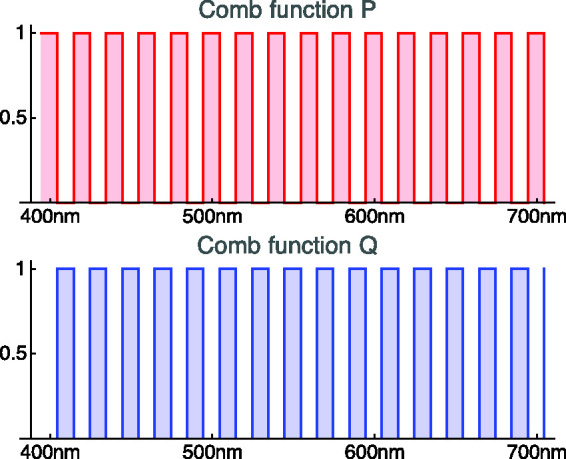
Two Dirac Comb Functions Having Fully Disjunct Spectral Footprints. There is no overlap at all. Nevertheless, each comb function uniformly covers the full range of the visual spectrum. That is because the spectral resolution of the human visual system is so low that the narrow gaps are irrelevant for most colorimetric computations. This renders the comb functions fit for “spectral multiplexing.”

**Figure C3.**
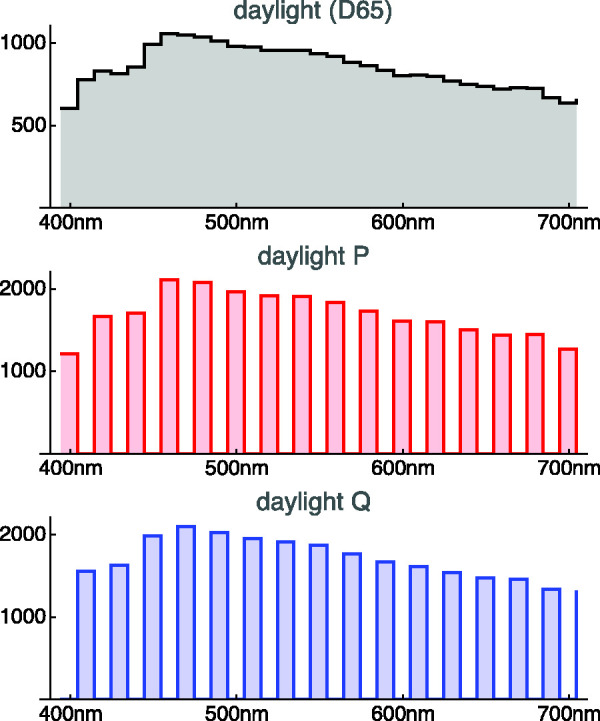
Three Metamers of cie d65 “Average Daylight.” At the top is the daylight spectrum itself. Notice that we use the 10-nm wide bins throughout. The two near metamers P and Q are two times the product of d65 and the corresponding comb function. In all relevant aspects, these illuminants are “white light,” not different from natural daylight d65. (The red and blue serve as convenient visual labels for the types P and Q.)

**Figure C4.**
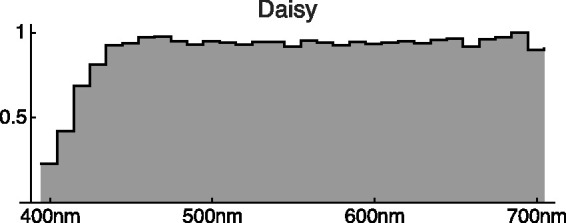
Daisy Spectral Reflectance Factor.

**Figure C5.**
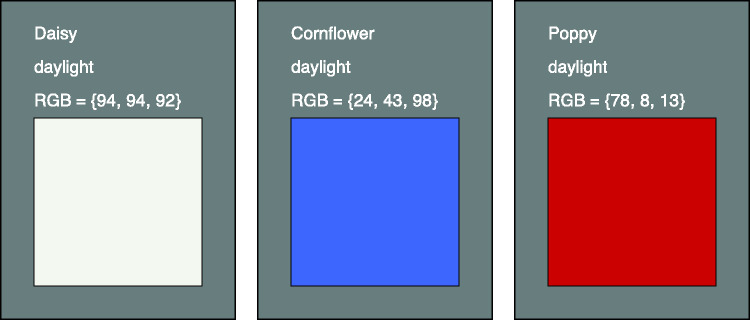
The Colors of the Daisy (*Bellis perennis*), the Cornflower (*Centaurea cyanus*), and the Poppy (*Papaver dubium*) Under Natural Daylight (cie d65) Illumination. RGB = red–green–blue.

**Figure C6.**
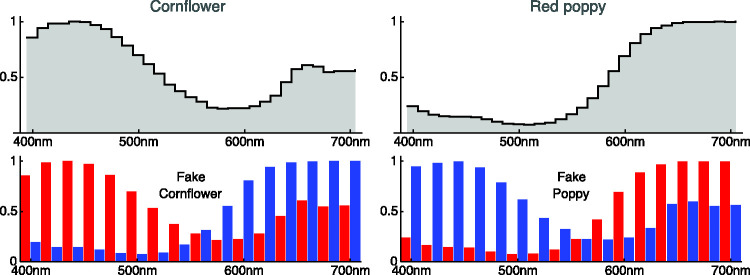
The Construction of the Fake Poppy and Fake Cornflower Spectral Reflectance Functions. The top row shows the reflectance spectra for the regular flowers. The bottom row shows the fake poppy and fake cornflower. (Notice that the P-type Dirac comb–function is shown in red, the Q-type in blue; compare Figures C2 and C3.)

**Figure C7.**
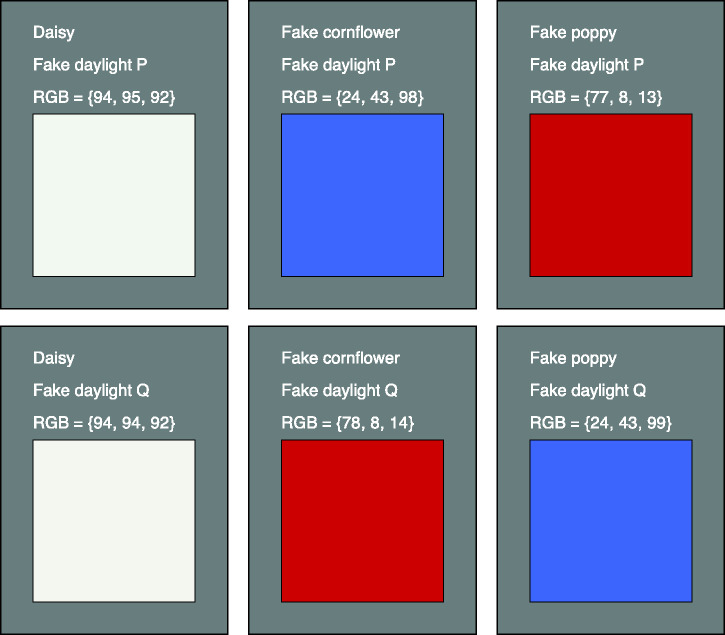
Renderings of the Fake Flowers (Cornflower Centre Column, Poppy Right Column) by the Two Weird Metamers (P, Top Row; Q, Bottom Row). Compare Figure 23. The rgb coordinate values have been calculated using the 10-nm bin width standard colorimetric methods. The fake flowers switch their hues as you change the illuminant. This might seem remarkable because the daisy (left column) is white under either illuminant. RGB = red–green–blue.

## Notes

1 We do not use explicit mathematical formalism in the text. This has been moved to a technical Appendix. The material in the appendices is sufficient to replicate all results mentioned in the text. For material readily found in the literature, we quote according to historical precedence and/or lucid exposition.

2 Conventionally known as the “Color Matching Matrix.”

3 One might prefer photon number density as a function of photon energy. It makes no essential difference. Here, we follow convention and do not even write “wavelength in vacuo.” Notice that physics limits radiant power density to the nonnegative quantities.

4 It is perhaps the most common way to put structure on a set. Given any equivalence relation, one immediately obtains a partition of the set.

5 In this article, we limit the discussion to the average generic observer. This makes sense because it includes the bulk of the population at the relevant level of accuracy.

6 The convexity is not trivial. For arbitrary cone sensitivities, it is common to violate convexity. Thus, whether the cone is indeed convex or not is an empirical issue. The accuracy by which the true cone spectra are known is much less than the accuracy of the color matching functions. These are available for a very broad spectral range, with an accuracy that is not even warranted by the psychometric data. The cie did much smoothing and interpolation. It is a moot point whether these data actually reflect cone action spectra in detail. Using this data, we find that the cone is indeed convex in the spectral range of 420 to 690 nm, but not in the extended range. There are violations of convexity lesser than 420 nm and greater than 690 nm. However, the contributions from these regions are so small that one may disregard them for all practical purposes. That is what we do here; thus, the spectrum cone is indeed convex *by selection*.

7 This is simply the abstract structure; the actual shape is discussed later. One perspective is the familiar spectrum locus in the cie xy chromaticity diagram. (As in Figure 4, left, which can also be read as Maxwell’s *hook*.)

8 Grassman refused to believe it. Newton had famously guessed it to be a full circle, although he did not really envisage the linear structure. Newton’s fame was the reason for Grassmann’s disbelief.

9 In the sense that it would not affect the null-space.

10 There are also cie rgb color matching functions. Here, the “RGB” refer to pragmatically selected monochromatic primaries. There is no formal relation to the rgb system of object colors as used in this article.

11 The system was set up in the era before computers, so it optimizes convenient hand, or slide-rule calculations, easy graphical representation, and so on. Nowadays, one might well optimize something else. It makes no essential difference.

12 Disregarding finite just noticeable differences, of course.

13 Newton and Grassmann apparently considered this so obvious that they did not consider it necessary to reveal their reasons for this feeling.

14 For instance, the closed, nonplanar polygonal arc yellow–green–cyan–blue–magenta–red in the rgb cube.

15 One actually has a choice to point out a color or a beam. There are many reasons to prefer the latter choice, as will become clear later in the article.

16 Velvet actually implements the whole idea. As radiation enters between the fibers that stand up from the surface, it has a hard time to come out again.

17 In fact, *much* better than the best “black paper,” which will certainly scatter at the percent level.

18 These are examples of optical identity changes on the short term, the time of psychogenesis, about 0.1 to 2 s. On longer time scales, such identity changes are very common. Think of the change from a caterpillar to a butterfly. Here, we really have visually fully distinct objects. On the short term, something very similar may happen as a *Morpho* only slightly moves its wings, or when a specular object suddenly reflects the sun toward your eye.

19 Northern skylight has been the standard illuminant of the artist’s studio for centuries.

20 The notion of *crate* is explained in another note. At this point, one should conceive of an infinitely dimensional parallelepiped. Its edge lengths are the spectral radiant power densities.

21 A *crate* is defined as a triple of vectors {a,b,c} (say), spanning a parallelepiped with vertices **o** (the origin), **a, b, c,** a+b, b+c, c+a and a+b+c. The crate that figures most prominently in this article is {{1,0,0},{0,1,0},{0,0,1}}, that is $I^{3}$, the unit cube.

22 It does not really make much difference. Many databases are limited to 400 to 700 nm; some colorimetric tables range from 320 to 830 nm. There is hardly anything lesser than 380 nm, or greater than 720 nm.

23 Indeed “natural” due to the overlap relations. For instance, yellow shares one part with red, another part with green, thus locating it between red and green.

24 We use γ=2.2 as a generic value in this article.

25 The matrix that transforms cie xyz coordinates to rgb coordinates (for cie d65) is: $\left(\begin{array}{ccc} +0.9286 & -0.2851 & -0.1472\\ -0.6437 & +1.0000 & +0.0444\\ +0.0630 & -0.0979 & +0.4431 \end{array}\right),$ where the absolute magnitude is irrelevant.

26 At least, to an excellent approximation. As noted before, minor excursions outside the cube are possible, albeit very rare.

27 With the difference that von Kries applied the mechanism to the retinal cone excitations, whereas we apply it to the red, green, and blue parts. Whereas our choice makes more sense from an engineering perspective, von Kries’s choice is a logical one for the physiologist. Of course, one may transform our choice to the basis used by von Kries, but then the adaptation involves a 3 × 3 matrix instead of 3 coefficients (which can be seen as the coefficients in a diagonal matrix). No big deal though, for we see no reason why a simple neural adaptation might not apply a full matrix scheme. The issue is evidently open to empirical investigation, but see Hirakawa and Parks (2005).

28 The Umwelt is the totality of anything that may enter by way of the sensors and anything that may be affected by the actuators (muscles, glands, . . .). The lifestyle includes the “soul” of the animal. For instance, although the Umwelts of the tiger and the lamb are not all that different, their lifestyles are very different indeed. This is discussed later in the text.

29 The empirical metric is complicated (inhomogeneous and anisotropic), but because it is needed in industrial applications, it has been suitably summarized as a numerical algorithm (Alman, 1993; CIE, 2004; Luo et al., 2001; Sharma et al., 2005). In practice, its applicability depends heavily on the context. Indeed, on the face of it, it seems most unlikely to us that such a metric, obtained from jnd measurements on very simple, artificial patterns, would generalize to more complicated patterns (as natural images) at all.

30 This database is due to Gegenfurtner’s laboratory at the University of Giessen (Germany). The pigments were obtained from Kremer Pigmente GmbH & Co. KG., located at Aichstetten (Germany). This database is the most extensive currently available on this type of spectral reflectance factors.

31 We select databases of spectral reflectance factors. Such databases are typically small, because measuring spectral reflection factors is time consuming. An alternative might seem to use hyperspectral image data, where there is no practical limit on the number of items. However, hyperspectral images yield the spectral radiance of scattered beams, not spectral reflectance factors. Using such data necessarily implies potentially problematic assumptions concerning the local spectral irradiant. That is why we decided against the use of hyperspectral image data.

32 From a physiological perspective, it would be natural when the von Kries-type adaptation contained a nonlinear part that would limit the result to the interior of the rgb cube. There is really no need to further speculate on such issues here.

33 The rgb chromaticity diagram derives from a projective transformation (much like that used in the case of the cie-*xy* diagram), defined by: $\{u,v\}=\left\{\frac{b-r}{w\sqrt{2}},\frac{2g-(b+r)}{w\sqrt{6}}\right\},\;\text{ where }\;w=r+g+b.$

34 On a Mid 2015 MacBook Pro, we generate about 2,000 instances per second with a generic Mathematica implementation.

35 The database at http://capbone.com/spectral-reflectance-database/ contains over seven million spectra.

36 On a Mid 2015 MacBook Pro, we generate about 1,000 instances per second with a generic Mathematica implementation.

37 One most conveniently generates fractals using the fast Fourier transform (FFT). In that case, one should realize that the FFT implies periodic signals. One should make sure to use a spectral range that is much larger than the visual range and cut away the outskirts at the conclusion of the computation. This is crucial to avoid systematic errors in the green–purple dimension. The same remark applies to a number of alternative algorithms, not relying on FFT.

38 On a Mid 2015 MacBook Pro, we generate about 600 metameric gray reflectance spectra per second.

39 Such boundary effects are to be expected because the regions near the spectrum limits have hardly any influence on color. All conventional algorithms (e.g., cofactor [or Laplace] expansion, or division-free row reduction) to compute null-space lead to similarly useless results.

40 The Hann function (Harris, 1978) is $w(n)=\sin^{2}\left(\frac{\pi n}{N-1}\right),\;\text{ for }\;n=0\cdots N-1.$

41 The whole procedure takes less than half a second in Mathematica. No doubt this could be speeded up significantly at the cost of extensive hand-coding in a language like c. Thus, it is certainly reasonable to attempt to investigate the influence of metamerism under ecological constraints in some detail.

42 That is why distinguishing marks from different modalities are to be preferred.

43 Because one has spatial and—when annotated—even semantic information.

44 The Lambert-W, or ProductLog function is of no further importance in our context. All we need is that $W(-1,\frac{1}{2\text{e}})\approx -2.67835\ldots$.

45 The formal proof is formulated in an abstract way; this is to stress the fact that the theorem is pure colorimetry and is fully independent of any phenomenological nonsense like “red” or “blue.” Abstraction is not to everybody’s liking. If so, continue at the paragraph *The proof dressed up in colorful garb* at the bottom of the next page. You won’t miss a thing.

## Appendix A: The Kubelka–Munk Theory of Scattering by Absorbing Turbid Layers

The Kubelka–Munk theory of radiative transport in turbid layers (Kubelka, 1948, 1954; Kubelka & Munk, 1931) is simple, but quite effective for paint layers, paper, cloth, and so forth. Here, we only need the simple case of optically thick layers. These have considerable covering power, as desirable in house paints, book paper, and cloth for underwear. Thick layers either cover their substrate (e.g., paints) or are at the boundary of extended volumes (e.g., mass colored plastics). In such cases, the reflectance of the turbid layer depends only upon the ratio of the absorption *K* and scattering *S* cross sections. That is because the geometry of the layer (its thickness) and the nature of the substrate (if any) become irrelevant.

The parameter that determines the diffuse reflectance of a thick layer depends only on the ratio *K*/*S*. This “spectral signature” *K*/*S* (Kortüm, 1969) is a true *material property*. That is why the spectral signature is widely used in materials research.

The spectral signature is related to the (observable!) reflectance factor *r* of an optically thick turbid layer as

(1) $$\frac{K}{S}=\frac{{\left(1-r\right)}^{2}}{2r},$$

its inverse is

(2) $$r=1+\frac{K}{S}-\sqrt{2\frac{K}{S}+{\left(\frac{K}{S}\right)}^{2}}$$

The spectral signature depends on physical quantities (scattering and absorption cross sections) that live in ${\mathbb{R}}^{+}$. This suggests that the appropriate physical domain is the logarithm of the spectral signature, that is log⁡(K/S).

Thus, we define the pair of (mutually inverse) functions

(3) $$f(r)=\log\left(\frac{{\left(1-r\right)}^{2}}{2r}\right)$$

(4) $$g(\rho )=-\sqrt{2e^{\rho }+e^{2\rho }}+e^{\rho }+1$$

where ρ=log⁡(K/S), the logarithm of the spectral signature.

The function f(r)) maps the unit interval I on the full affine line A, and the inverse function g(ρ) maps the full line to the interval (Figure 12).

This pair of mutually inverse functions allows homomorphic filtering of spectral reflectance factors.

For instance, it is the correct way to do PCA on reflectance databases.

## Appendix B: A Formal, Generic Model of Spectral Reflectance

In a simple model of generic physics, one assumes that spectral articulation is the result of various (perhaps many) mutually independent physical causes. Each process imprints a local structure on the spectrum at some random location.

The spectral width of the local structures is the relevant property. For simplicity, one considers just a single width.

The distribution of locations is assumed to be random and uniformly distributed. This makes it possible to derive very simple relations that will hold “on the average.”

A suitable model of a local structure is a Laplace pulse (Figure B1, left):

(5) $$p(x,x_{0},\tau )=\pm \frac{{\text{e}}^{-\frac{\left|x-x_{0}\right|}{\tau }}}{2\tau }$$

where *x*_0_ denotes the location, and where the width at half height is 2τlog⁡2≈1.386τ. Its Fourier transform is a Cauchy, or Lorenz, distribution. A random superposition of many of such pulses yields a fractal signal with power spectrum

(6) $$S(f,\tau )=\frac{2\sqrt{\frac{2}{\pi }}\tau }{{\left(1+f^{2}\tau^{2}\right)}^{2}}$$

which falls off with the inverse fourth power from the frequency $f_{0}=1/\tau$.

The corresponding autocorrelation function is (Figure B1, right)

(7) $$R(x,\tau )=\frac{e^{-\frac{\left|x\right|}{\tau }}(\left|x\right|+\tau )}{\tau },$$

which has a width at half height of

(8) $$\Delta x=-2\tau \left[1+W(-1,\frac{1}{2\text{e}})\right]\approx 3.36\tau$$

where the function *W*(*k*, *z*) is the Lambert-W, or ProductLog function.^44^

The halfwidth of the autocorrelation function is the empirically relevant parameter. The model allows one to estimate it from the articulation power spectrum. This spectrum can be observed as a statistical property of reflectance databases. The parameter specifies the expected smoothness of instances from the database. This can be exploited to design a generative model. This model allows one to construct artificial spectra that cannot be distinguished from actual database members.

Such generative models are required to overcome the problem that existing databases tend to be rather limited. They allow statistical extrapolation.

## Appendix C: Spenser’s Roses Red, and Violets Blew Is Nonsense

*Roses are red, violets are blue* is a folk wisdom that has been around for centuries (Spenser, 1590). But does it make sense? In some societies, the belief in the efficaciousness of rain dancing is another folk wisdom that has been around for centuries. Its venerable age does not render that belief true. In the case of the colors, philosophy disagrees; it may amuse the poet to speak of *mental paint*, but the scientist should know better (Byrne & Hilbert, 2003; Gatzia, 2010; Hardin, 1988). *Colors are not object properties*. (What that means is illustrated in Figure C1.)

The philosophers are right about that. Here, we set out to provide a simple formal proof that ought to be convincing as a demo.^45^

### Abstract Theorem in Colorimetry

We state the (really trivial) theorem in a few successive stages. The abstraction effectively hides the fact that the formalism is interesting when interpreted as *being about something*. This makes it simpler to notice that the theorem is indeed trivial. Once dressed up with colorful interpretations, it appears near to magical; we do that later.

#### Setting

Consider a formal colorimetry. It boils down to the definition of a function and of a condition. The condition imposes an equivalence relation on the domain of the function.

Consider a linear function P:B↦ℂ (with $\mathbb{C}={\mathbb{R}}^{3}$) from the space B of real functions F(λ) of a real variable λ∈I on the finite interval $I\subset {\mathbb{R}}^{+}$. The functions are restricted to being nonnegative and bounded, for all λ∈I we have 0≤F(λ)<∞. Linearity implies P (αF+βG)=αP F+βP G.

The condition is called “equivalence.” When P F≡P G, we say that F∼G, or “*F* and *G* are equivalent,” implying that *F* and *G* may be exchanged in formal expressions (Grassmann, 1853).

This completely characterizes a formal colorimetry, for instance, cie xyz. There are infinitely many, mutually equivalent, implementations possible.

In addition, we consider maps R:B↦B. A map **R** is implemented through a function R(λ) with values on the unit interval $I^{1}$ such that G=RF is obtained by pointwise multiplication, that is G(λ)=R(λ)F(λ).

So much for the setting; now we get to the theorem:

#### Context

There are specified a special function D(λ)>0 and two maps $R_{A},\;R_{B}$ such that $PR_{A}D≁PR_{B}D$. **Theorem** One may construct functions P(λ)≥0 and Q(λ)≥0 and maps ${\bar{R}}_{A}$ and ${\bar{R}}_{B}$ such that PP∼PQ∼PD and such that $P{\bar{R}}_{A}P∼P{\bar{R}}_{B}Q$ and $P{\bar{R}}_{B}P∼P{\bar{R}}_{A}Q$ for arbitrary elements $P{\bar{R}}_{A}P$ and $P{\bar{R}}_{A}Q$ of ℂ.

#### Consequence

One might perhaps expect that the distinct points $\left\{PR_{A}D,PR_{B}D\right\}$ in ℂ might allow one to distinguish between A and B, but that is wrong because of the theorem. The error occurs because *P*, *Q* are confused with *D* (and thus with each other) because PP∼PQ∼PD. The interpretation (see later) is that “colors are not object properties.”

#### Partial translation (not part of the theorem!)

Here is the intended interpretation: The functions F(λ) are radiant power spectra. The parameter *λ* is the wavelength on the interval I, roughly the range 390 to 710 nm. The linear operator **P** is implemented by the color matching functions. The space ℂ is “color space,” its elements are “colors.” The maps **R** are the material properties (the function R(λ) the spectral reflectance factor). The special function *D* is the daylight spectrum. An entity like PRD∈ℂ is an “object color.” Because the color matching functions and the daylight spectrum are fixed, object colors are determined by their spectral reflectance functions. Maps $R_{A},R_{B}$ are material properties possessed by objects A,B (say). The consequence says that colors A and B (say “red” and “blue”) may sometimes (“sometimes” meaning: in the absence of knowledge about the illuminant, except for its rendering of the white standard) appear as B and A (that would be “blue” and “red”) despite the fact that “white” always looks “white.” *Ergo*, colors A and B cannot correspond to objects A and B.

### The Proof Dressed Up in Colorful Garb

The proof is one by construction. Because the abstract formalism of colorimetry tends to mask the meaning for many people, we “dress up” the proof with some colorful interpretation. Such interpretations are meaningless as the formalism of colorimetry goes but are likely to aid in the understanding of what goes on.

Colorimetry is not just a formalism. It is only of interest because it is *about* facts that involve physics, objects, the luminous environment, and observations. The formal proof can be obtained from the following description by stripping it from all interpretation if one desires to do so. It will reduce it to a correct, but meaningless formal triviality. The interpretation is of interest, the formal theorem hardly.

We propose to set up two fake flowers and two fake daylights such that we will obtain the remarkable result suggested in Figure C1.

We set up colorimetry for a visual range of 390 to 710 nm, sampled in 10-nm wide bins. This makes it possible to show graphically how the trick is done. Remember that the bin width is arbitrary. Thus, the trick still works in the limit to arbitrarily narrow bin width. Then, the articulations would certainly go unnoticed.

Our main tool in implementing the trick is to define two Dirac comb functions that have no spectral overlap, yet both cover the full spectral range (Figure C2) uniformly, that is, up to the existence of narrow gaps. The gaps can be made arbitrarily narrow, so they will never show up in colorimetric calculations. But even rather finite gaps will do the trick in practice. In the example, 10 nm proves to be narrow enough for a convincing demo. (Our examples use that to do the colorimetric computations.)

What is crucial here is that the product of these two comb functions is always identically zero, whereas any comb function becomes effectively just a constant (thus, the two comb functions become effectively identical!) in colorimetric relations when the sample rate becomes arbitrarily high.

A first use of the comb functions is to define two weird metamers of cie d65 “average daylight” (see Figure C3.) These are indeed “weird” spectra because of the numerous gaps. We make up for the gaps by increasing the spectral radiance by a factor of two. Thus, the total radiant power of all three beams is the same. These weird sources work out just like the standard daylight in almost any realistic setting. There is nothing visually weird about them.

We also define some weird flower spectral reflectance factors. To do that, we start from regular flower reflectance distributions.

As this is just a formal proof, there is no essential need for actual flower spectra. Of course, this really makes no difference to the proof. However, it is perhaps more convincing, because it renders the example less “artificial.”

Unfortunately, we could not find suitable rose and violet spectra to illustrate Spenser’s poem. Thus, we settled for three items from the very convenient Floral Reflectance Database (Arnold et al., 2010), namely (see Figure C5):

**ID3126** White daisy, *Bellis perennis* (Innsbruck, Austria; flower tip, upper side);

**ID2537** Blue cornflower, *Centaurea cyanus* (Amoeneburg, Germany; bell shape, inner side);

**ID2112** Red poppy, *Papaver dubium* (Strausberg, Germany; whole flower, upper side).

The daisy was included as a white reference; it has a very flat spectrum (Figure C4). The cornflower and the poppy show very vivid blue and red colors in normal daylight (Figure C5).

We cut the spectral range (the Floral Reflectance Database is focussed on insect vision, so it includes the near ultraviolet) and subsampled to the coarse 10-nm bins. Then, we normalized the maximum spectral reflectance factor to one. That is not necessary but makes it easier to effectively show the calculated rgb colors.

From each flower reflectance, we derive two metameric versions, by multiplying them with the Dirac comb functions. Under natural daylight, these appear as shades, because the total reflectance is cut into half due to the gaps. But the radiance of the weird daylights makes up for that. A weird flower illuminated by a weird daylight of the same type (either P or Q) looks exactly the same as the regular flower under natural daylight. Illuminated by a weird daylight of the opposite type it will look black, because then the illuminant will not be reflected at all.

Now for the most essential part of the trick (see Figure C6). We combine the P-type poppy with the Q-type cornflower. This yields a spectral reflectance factor that will yield a purple shade (one half red plus one half blue) as illuminated with natural daylight. But illuminated with a P-type Dirac comb function, it will show only the red of the poppy, whereas illuminated with a Q-type Dirac comb function, it will show only the blue of the cornflower.

Thus, the weird daylight metamers let us *select* either the poppy or the cornflower. It is the crux of the proof.

Of course, we can as well do things the other way around. We combine the Q-type poppy with the P-type cornflower. This again yields a spectral reflectance factor that will yield a purple shade (one half red plus one half blue) as illuminated with natural daylight. But illuminated with a P-type Dirac comb function, it will show only the blue of the cornflower, whereas illuminated with a Q-type Dirac comb function, it will show only the red of the poppy.

Thus, swapping the P- and Q-type metamers of daylight allow us to swap the colors of the poppy and the cornflower. This is remarkable, because both fake daylights are—like regular average daylight—“white light.” This is visually obvious by checking the color of the daisy. The daisy reflectance factor is approximately unity all over the visual range. Thus, the daisy (of course) looks white under natural daylight. But it *looks equally white under each of the fake daylights*. This is a “visual proof” that all these illuminants are “white light,” which, in that sense, they are.

As a result, purely visually, one has a case of sheer magic (Figure C7). We have constructed a formal model that achieves precisely what we faked (by photoshopping) in Figure C1. It is indeed possible that an otherwise invisible change of illuminant lets red poppies and blue cornflowers appear as blue poppies and red cornflowers. This proves that red is not a property of the poppy, nor blue a property of the cornflower.

Formally, we effectively implemented *spectral multiplexing*. A mean trick perhaps, but it works. In the general case, there are no constraints, except for the usual physical constraints. The spectral multiplexing is a very general technique. Instead of red and blue, we might have taken *any* two colors, even *any number* of colors.

The crucial factors that render this possible are, first, that one accepts highly articulated spectra (for this demonstration, we used comb functions with a bin width of 10 nm), and, second, that such articulations are perfectly synchronized between radiant and reflectance spectra.

It is especially the latter factor that is crucial. The spectral articulations are essentially *hidden variables* because they are lower than the resolution of the human visual system. But object colors involve an essentially nonlinear process, the spectrally selective scattering of the illumination by the object. Roughly, the spectrum scattered to the eye is the product of the illuminant radiant spectrum I(λ) and the spectral reflectance factor R(λ) of the object. The weird illuminants involve multiplication with the comb–functions P(λ) and Q(λ) and similar for the weird reflectances. Thus, the fake poppy has reflectance $P(\lambda )R_{\text{poppy}}(\lambda )+Q(\lambda )R_{\text{cornflower}}(\lambda )$. Then, illuminated with the weird daylight of the P-type, we obtain (here, we omit the dependence on *λ* for clarity):

$$(PI)(PR_{\text{poppy}}+QR_{\text{cornflower}})=P^{2}IR_{\text{poppy}}+\mathrm{PQI}R_{\text{cornflower}}=IR_{\text{poppy}}$$

because $P^{2}(\lambda )=1$ and P(λ)Q(λ)=0. Likewise, illuminated with the weird daylight of the Q-type, we obtain:

$$(QI)(PR_{\text{poppy}}+QR_{\text{cornflower}})=\mathrm{QPI}R_{\text{poppy}}+Q^{2}IR_{\text{cornflower}}=IR_{\text{cornflower}}$$

because $Q^{2}(\lambda )=1$ and Q(λ)P(λ)=0.

It is as simple as that. The multiplication reveals the hidden variables, whereas the high-resolution articulation renders them “hidden” with respect to all linear colorimetric computations.

Although highly articulated spectra are physically possible (e.g., pressed didymium glass powders), such correlations are extremely singular (e.g., the sodium d-doublet happens—by sheer accident—to be selectively absorbed by a didymium glass filter).

So we currently lack the appropriate technology to make the magic work in the laboratory. But there is nothing in physics that would forbid the possibility.

Finally, here are a few useful corollaries of the theorem we just proved. They follow immediately, so one needs to do no additional derivations.

**Repeated views**: The color of an object seen once does not predict the color of the object seen on the next occasion;

**Comparison**: When two objects have the same color, it does not follow that they will look the same on revisiting;

**Achromaticity**: Being colorless is meaningless. Any object may look colorless at times, and any colorless object may look colored at times; and

**Object specificity**: An object may appear in any color at certain times.

All these statements represent an attack of folk wisdoms. All these wisdoms are proven wrong. That should give one something to ponder. One wonders what philosophers would make of it.

Notice that this puts the blame on the objects, not on the sources. This seems reasonable from a phenomenological perspective. After all, the sources are just “white light.” One can easily demonstrate that with a piece of white paper. Yet that idea is quite wrong, of course. The effects are due to nonlinear interactions between hidden variables in the illuminant with hidden variables in the objects. If one puts the blame on the objects, it is simply because the light is considered beyond suspicion. That might seem to be the case because the light is considered “given.” It is the same for all objects. In that respect, the *light* is not unlike space (as a *container*), or time (as flowing in its own sweet way). But space, time and light are nothing but implicit *assumptions*. Finally, naive persons are not likely to accept that colors are not object properties and that they have been deluded in this all their lives. They smell a fish and would rather go with the folk wisdom. Such intuitions derive from our ancestral core as tundra hunter-gatherers.

Evolution does not make us wise but tunes us to efficacious interactions—given Umwelt, lifestyle and ecological niche.

That colors are not object properties is indeed *formally true*. However, that fact is also *pragmatically irrelevant* because the conditions under which it might become apparent are extremely unlikely in the ecological setting.

Complicated spectral articulations are very rare—even absent—in the tundra hunter-gatherer niche. Correlations of spectral articulations between illuminant and reflectance spectra are so unlikely as to be effectively nonexistent. The reason for that is that there are no known physical processes that would bring such correlations about, whereas pure change needs hardly be reckoned with.

## References

[bibr1-2041669520958431] Albertazzi L. (2013). Experimental phenomenology: An introduction In: Albertazzi L. (Ed.), Handbook of experimental phenomenology: Visual perception of shape, space and appearance (pp. 1–37). John Wiley & Sons.

[bibr2-2041669520958431] Albertazzi L. Koenderink J. Doorn A. van (2015). Chromatic dimensions earthy, watery, airy, and fiery. Perception, 44, 1153–1178. 26562887 10.1177/0301006615594700

[bibr3-2041669520958431] Alman D. (1993). Industrial color difference evaluation. Color Research and Application, 18, 137–139.

[bibr4-2041669520958431] Arkhangelskii A. V. Pontryagin L. S. (1990). General topology I. Springer–Verlag.

[bibr5-2041669520958431] Arnold S. E. J. Faruq S. Savolainen V. McOwan P. W. Chittka L. (2010). FReD: The Floral Reflectance Database – A web portal for analyses of flower colour. PLoS One, 5(12), e14287 10.1038/npre.2008.1846.1 21170326 PMC3000818

[bibr7-2041669520958431] Bouma P. J. (1946). Kleuren en kleurenindrukken [Colors and color sensations]. Philips Technische Bibliotheek, Meulenhoff & Co. N.V.

[bibr8-2041669520958431] Brouwer L. E. J. (1912). Über Abbildungen von Mannigfaltigkeiten [On maps of manifolds]. Mathematische Annalen, 71, 97–115.

[bibr9-2041669520958431] Brualdi R. A. (2004). Introductory combinatorics (4th ed.). Pearson.

[bibr10-2041669520958431] Byrne A Hilbert D. R. (2003). Color realism and color science. Behavioral and Brain Sciences, 26, 3–64. 14598439 10.1017/s0140525x03000013

[bibr11-2041669520958431] Centore P. (2017). The geometry of colour. Paul Centore.

[bibr12-2041669520958431] Chiao C.-C. Cronin T. W. Osorio D. (2000). Color signals in natural scenes: Characteristics of reflectance spectra and effects of natural illuminants Journal of the Optical Society of America. A, 17(2), 218–224. 10.1364/josaa.17.000218 10680623

[bibr13-2041669520958431] Commission Internationale de l’Éclairage. (1932). Commission Internationale de lÉclairage proceedings 1931. Cambridge University Press.

[bibr14-2041669520958431] Commission Internationale de l’Éclairage. (2004). A colour appearance model for colour management systems: CIECAM02 (Publication 159). Bureau Central de la CIE.

[bibr15-2041669520958431] Farnsworth D. (1943). The Farnsworth–Munsell 100–hue and dichotomous tests for color vision. Journal of the Optical Society of America, 33, 568–574.

[bibr16-2041669520958431] Feynman R. Leighton R. Sands M. (1964–1966). *The Feynman lectures on physics (3 volumes)* (Library of Congress Catalog Card No. 63-20717). Addison-Wesley Publishing Company.

[bibr18-2041669520958431] Frege G. (1892). Über Sinn und Bedeutung [On sense and reference]. Zeitschrift für Philosophie und philosophische Kritik, 100, 25–50.

[bibr19-2041669520958431] Friel M. (2010). Still life painting atelier: An introduction to oil painting (p. 20). Watson-Guptill.

[bibr20-2041669520958431] Gatzia D. E. (2010). Colour fictionalism. Rivista Di Estetica, 1(43), 109–123.

[bibr21-2041669520958431] Gegenfurtner K. R. Rieger J. (2000). Sensory and cognitive contributions of color to the recognition of natural scenes. Current Biology, 10(13), 805–808. 10898985 10.1016/s0960-9822(00)00563-7

[bibr22-2041669520958431] Gibbons A. (2006). The first human: The race to discover our earliest ancestors (1st ed.). Doubleday.

[bibr23-2041669520958431] Gilchrist A. Kossyfidis C. Bonato F. Agostini T. Cataliotti J. Li X. J. Shehar B. Annan V. Economou E. (1999). An anchoring theory of lightness perception. Psychological Review, 106(4), 795–834. 10560329 10.1037/0033-295x.106.4.795

[bibr24-2041669520958431] Goethe J. W. (1810). Zur Farbenlehre [Theory of colors]. Cotta.

[bibr25-2041669520958431] Grassmann H. (1853). Zur Theorie der Farbenmischung [On the theory of color mixtures]. Annalen der Physik und Chemie, 165(5), 69–84.

[bibr26-2041669520958431] Griffin L. D. (2019). Reconciling the statistics of spectral reflectance and colour. PLoS One, 14(11), e0223069 10.1371/journal.pone.0223069 31703060 PMC6839875

[bibr27-2041669520958431] Hardin C. L. (1988). Color for philosophers. Hackett.

[bibr28-2041669520958431] Harris F. J. (1978). On the use of windows for harmonic analysis with the discrete Fourier transform. Proceedings of the IEEE, 66(1), 51–83.

[bibr29-2041669520958431] Hering E. (1905–1911). *Grundzüge der Lehre vom Lichtsinn* [Outlines of a Theory of the Light Sense]. Sonderabdr. a. d. Hdb. d. Augenheilkunde. Voss.

[bibr30-2041669520958431] Hirakawa K. Parks T. W. (2005, October). *Chromatic adaptation and white-balance problem* [Paper presentation]. Proceedings/ICIP International Conference on Image Processing 3: III (pp. 984–987). Piscataway, NJ, United States.

[bibr31-2041669520958431] Hubbe M. A. Pavlak J. J. Koukoulas A. A. (2008). Paper’s appearance: A review. BioResources, 3(2), 627–665.

[bibr32-2041669520958431] Jaynes E. T. (1968), Prior probabilities. IEEE Transactions on Systems Science and Cybernetics, SSC*-* 4(3), 227–241.

[bibr33-2041669520958431] Jaynes E. T. (1973), The well-posed problem. Foundations of Physics, 3, 477–493.

[bibr34-2041669520958431] Joshi J. J. Vaidya D. B. Shah H. S. (2001). Application of multi-flux theory based on mie scattering to the problem of modeling the optical characteristics of colored pigmented paint films. Color Research and Application, 26(3), 234–245.

[bibr35-2041669520958431] Judd D. B. Wyszecki G. (1975). Color in business, science and industry. Wiley.

[bibr36-2041669520958431] Koenderink J. (2010a). Colour for the sciences. MIT Press.

[bibr37-2041669520958431] Koenderink J. (2010b). The prior statistics of object colors. Journal of the Optical Society of America A, 27(2), 206–217. 10.1364/JOSAA.27.000206 20126232

[bibr38-2041669520958431] Koenderink J. (2018a). The colours and the spectrum In: MacDonald L. W. Biggam C. P. Paramei G. V. (Eds.), Progress in colour studies: Cognition, language and beyond (Chapter 1, pp. 5–22). John Benjamins.

[bibr39-2041669520958431] Koenderink J. (2018b). Colour in the wild. de Clootcrans Press.

[bibr40-2041669520958431] Koenderink J. Doorn A. van (2017). Colors of the sublunar. i–Perception, 8(5), 1–30. 10.1177/2041669517733484 PMC5624368 28989697

[bibr41-2041669520958431] Koenderink J. Doorn A. van Ekroll V. (2016). Color picking: The initial 20s. ACM Transactions on Applied Perception, 13(3), Article 13, 1–25.

[bibr42-2041669520958431] Koenderink J. Doorn A. van Gegenfurtner K. (2018). Graininess of RGB–display space. i–Perception, 9(5), 1–46. https://journals.sagepub.com/doi/pdf/10.1177/2041669518803971 10.1177/2041669518803971 PMC6231535 30430002

[bibr43-2041669520958431] Kohonen O. Parkkinen J. Jääskeläinen T. (2006). Databases for spectral color science. Color Research and Application, 31(5), 381–390.

[bibr44-2041669520958431] Kortüm G. (1969). Reflectance spectroscopy. Springer-Verlag.

[bibr45-2041669520958431] Koschmieder H. (1925). Theorie der horizontalen Sichtweite [Theory of horizontal visibility]. Beiträge zur Physik der freien Atmosphäre, 12, 33–55, 171–181.

[bibr46-2041669520958431] Kubelka P. (1948). New contributions to the optics intensely light- scattering materials. Part I. Journal of the Optical Society of America, 38, 448–457. 18916891 10.1364/josa.38.000448

[bibr47-2041669520958431] Kubelka P. (1954). New contributions to the optics intensely light- scattering materials. Part II: Nonhomogeneous layers. Journal of the Optical Society of America, 44, 330–335. 10.1364/josa.38.000448 18916891

[bibr48-2041669520958431] Kubelka P. Munk F. (1931). Ein Beitrag zur Optik der Farbanstriche [On the optics of paint layers]. Zeitschrift für Technische Physik (Leipzig), 12, 593–601.

[bibr49-2041669520958431] Kuehni R. G. (2004). Variability in unique hue selection: A surprising phenomenon. Color Research and Application, 29(2), 158–162.

[bibr50-2041669520958431] Küppers H. (1978). Das Grundgesetz der Farbenlehre [The basis of color theory]. DuMont.

[bibr51-2041669520958431] Küppers H. (1989). Harmonielehre der Farben: Theoretische Grundlagen der Farbgestaltung [Color Harmony: Theoretical basis of color design]. DuMont.

[bibr52-2041669520958431] Küppers H. (2005). Einführung in die Farbenlehre [Introduction to color theory]. DuMont.

[bibr53-2041669520958431] Lenz R. (2002). Two stage principal component analysis of color. IEEE Transactions on Image Processing, 11(6), 630–635. 18244661 10.1109/TIP.2002.1014994

[bibr54-2041669520958431] Linhares J. M. M. Pinto P. D. Nascimento S. M. C. (2008). The number of discernible colors in natural scenes. Journal of the Optical Society of America A, 25(12), 2918–2924. 10.1364/josaa.25.002918 19037381

[bibr56-2041669520958431] Luo M. R. Cui G. Rigg B. (2001). The development of the CIE 2000 colour difference formula:CIEDE2000. Color Research & Application, 26, 340–350.

[bibr57-2041669520958431] MacAdam D. L. (1942). Visual sensitivities to color differences in daylight. Journal of the Optical Society of America, 32, 247–274. 10.1364/josa.39.000808 18142394

[bibr58-2041669520958431] MacAdam D. L. (1947). Note on the number of distinct chromaticies. Journal of the Optical Society of America, 37, 308–309. 20295356 10.1364/josa.37.0308_1

[bibr59-2041669520958431] Maloney L. T. (1986). Evaluation of linear models of surface spectral reflectance with small numbers of parameters. Journal of the Optical Society of America A, 3(10), 1673–1683. 10.1364/josaa.3.001673 3772629

[bibr60-2041669520958431] Marin-Franch I. Foster D.H. (2010). Number of perceptually distinct surface colors in natural scenes. Journal of Vision, 10(9), 9. 10.1167/10.9.9 20884607

[bibr61-2041669520958431] Maxwell J. C. (1855). Experiments on colour, as perceived by the eye, with remarks on colour blindness. Transactions of the Royal Society of Edinburgh, 21, 275–289.

[bibr62-2041669520958431] Morimoto T. Kishigami S. Linhares J. M. M. Nascimento S. M. C. Smithson H. E. (2019). Hyperspectral environmental illumination maps: Characterizing directional spectral variation in natural environments. Optics Express, 27, 32277–32293. 31684444 10.1364/OE.27.032277 PMC7028397

[bibr63-2041669520958431] Munsell A. H. (1905). A color notation. H. H. Ellic Co.

[bibr64-2041669520958431] Munsell A. H. (1912). A pigment color system and notation. The American Journal of Psychology, 23(2), 236–244.

[bibr65-2041669520958431] Nascimento S. M. C. Ferreira F. P. Foster D. H. (2002). Statistics of spatial cone-excitation ratios in natural scenes. Journal of the Optical Society of America A, 19(8), 1484–1490. 10.1364/josaa.19.001484 PMC1965492 12152688

[bibr66-2041669520958431] Newton I. (1704). Opticks: Or, a treatise of the reflexions, refractions, inflexions and colours of light. Sa.Smith and Benj.Walford.

[bibr67-2041669520958431] Noorlander C. Koenderink J. J. (1983). Spatial and temporal discrimination ellipsoids in color space. Journal of the Optical Society of America, 73(11), 1533–1543. 6644399 10.1364/josa.73.001533

[bibr68-2041669520958431] Olkkonen M Hansen T. Gegenfurtner K. R. (2009). Categorical color constancy for simulated surfaces. Journal of Vision, 9(12), 6.1–6.18. 10.1167/9.12.6 20053097

[bibr69-2041669520958431] Oppenheim A. V. Schafer R. W. Stockham T. G. (1968). Nonlinear filtering of multiplied and convolved signals. Proceedings of the IEEE, 56(8), 1264–1291.

[bibr70-2041669520958431] Ostwald W. (1917). Die Farbenfibel [Guide to color]. Unesma.

[bibr71-2041669520958431] Ostwald W. (1919). Einführung in die Farbenlehre [Introduction to color theory]. Unesma.

[bibr72-2041669520958431] Pauler N. (2001). Paper optics. AB Lorentzen & Wettre.

[bibr73-2041669520958431] Pennebaker W. B. Mitchell J. L. (2004). JPEG still image data compression standard (eighth printing). Kluwer.

[bibr74-2041669520958431] Pilorget C. Fernando J. Ehlmann B. L. Schmidt F. Hiroi T. (2016). Wavelength dependence of scattering properties in the vis–nir and links with grain-scale physical and compositional properties. Icarus, 267, 296–314.

[bibr75-2041669520958431] Pointer M. R. Attridge G. G. (1998). The number of discernible colours. Color Research and Application, 23(1), 52–54.

[bibr76-2041669520958431] Poynton C. A. (1993). Gamma and its disguises. Journal of the Society of Motion Picture and Television Engineers, 102(12), 1099–1108.

[bibr77-2041669520958431] Quiller S. (1989). Color choices: Making color sense out of color theory. Watson-Guptill.

[bibr78-2041669520958431] Runge P. O. (1810). Die Farben-Kugel, oder Construction des Verhältnisses aller Farben zueinander [Color Sphere, the construction of all mutual relations of colors]. Perthes.

[bibr79-2041669520958431] Schopenhauer A. (1816). Ueber das Sehn und die Farben: eine Abhandlung [An essay on vision and colors] (2nd ed.). Hartknoch.

[bibr80-2041669520958431] Schrödinger E. (1920). Theorie der Pigmente von größter Leuchtkraft [Theory of pigments of highest intensity]. Annalen der Physik, 4(62), 603–622.

[bibr81-2041669520958431] Schuster A. (1905). Radiation through a foggy atmosphere. Astrophysical Journal, 21(1), 1–22.

[bibr82-2041669520958431] Sharma G. Wu W. Dalal E. N. (2005). The CIEDE2000 color-difference formula: Implementation notes, supplementary test data, and mathematical observations. Color Research & Applications, 30, 21–30.

[bibr83-2041669520958431] Smith A. R. Lyons E. R. (1996). HWB – A more intuitive hue-based color model. Journal of Graphics Tools, 1(1), 3–17.

[bibr84-2041669520958431] Smith T. Guild J. (1931– 1932). The C.I.E. colorimetric standards and their use. Transactions of the Optical Society, 33, 73–134.

[bibr85-2041669520958431] Spenser E. (1590). The Faerie Queene. William Ponsonby.

[bibr86-2041669520958431] Stenius Å. S. (1951a). The application of the Kubelka-Munk theory to the diffuse reflection of light from paper, I. Svensk Papperstidning, 54, 663–670.

[bibr87-2041669520958431] Stenius Å. S. (1951b). The application of the Kubelka-Munk theory to the diffuse reflection of light from paper, II. Svensk Papperstidning, 54, 701–709.

[bibr88-2041669520958431] Stenius Å. S. (1953). The application of the Kubelka-Munk theory to the diffuse reflection of light from paper, III. Svensk Papperstidning, 56, 607–614.

[bibr89-2041669520958431] Strutt J. (Lord Rayleigh) (1899). On the transmission of light through an atmosphere containing small particles in suspension, and on the origin of the blue of the sky. Philosophical Magazine, 5(47), 375–394.

[bibr90-2041669520958431] Tellex P. A., & Waldron J. R. (1955). Reflectance of magnesium oxide. Journal of the Optical Society of America, 45(1), 19.

[bibr91-2041669520958431] Tinbergen N. (1963). On aims and methods of ethology. Zeitschrift für Tierpsychologie, 20(4), 385–516.

[bibr92-2041669520958431] Van den Akker J. A. (1949). Scattering and absorption of light in paper and other diffusing media. Tappi Journal, 32, 498–501.

[bibr93-2041669520958431] van Esch J. A. Koldenhof E. E. Doorn A. van Koenderink J. J. (1984). Spectral sensitivity and wavelength discrimination of the human peripheral visual field. Journal of the Optical Society of America A, 1(5), 443–450. 10.1364/josaa.1.000443 6726492

[bibr94-2041669520958431] von Helmholtz H. (1855). Ueber die Zusammensetzung von Spectralfarben [On compositions of spectral colors]. Annalen der Physik und Chemie, 94, 1–28.

[bibr95-2041669520958431] von Helmholtz H. (1867). Handbuch der physiologischen Optik [Handbook of Physiological Optics]. Voss.

[bibr96-2041669520958431] von Helmholtz H. (1891). *Versuch einer erweiterten Anwendung des Fechnerschen Gesetzes im Farbensystem* [Attempt of a generalization of Fechner's Law in color space]. Zeitschrift für Psychologie und Physiolologie der Sinnesorgane, 2, 1–30.

[bibr97-2041669520958431] von Helmholtz H. (1892a). Versuch, das psychophysische Gesetz auf die Farbenunterschiede trichromatischer Augen anzuwenden [Attempt to apply Fechner's Law to trichromatic vision]. Zeitschrift für Psychologie und Physiologie der Sinnesorgane, 3, 1–20.

[bibr98-2041669520958431] von Helmholtz H. (1892b). *Kürzeste Linien im Farbensystem* [Geodesics in color space]. Zeitschrift für Psychologie und Physiololgie der Sinnesorgane, 4, 108–122.

[bibr99-2041669520958431] von Kries J. (1905). Die Gesichtsempfindungen [Vision]. In Nagel W. (Ed.), Handbuch der Physiologie der Menschen 3 (pp. 109–282). Vieweg, Braunschweig.

[bibr100-2041669520958431] von Uexküll J. (1926). Theoretical biology. Harcourt.

[bibr101-2041669520958431] von Uexküll J. (2010). *A foray into the worlds of animals and humans: With a theory of meaning* (Joseph D. O’Neil, Trans.). University of Minnesota Press.

[bibr102-2041669520958431] Witzel C. Gegenfurtner K. R. (2018). Color perception: Objects, constancy, and categories. Annual Review of Vision Science, 4, 475–499. 10.1146/annurev-vision-091517-034231 30004833

[bibr103-2041669520958431] Wright W. D. Pitt F. H. G. (1934). Hue discrimination in normal colour vision. Proceedings of the Physical Society, 46, 459–473.

[bibr104-2041669520958431] Wyszecki G. Stiles W. S. (1967). Color science: Concepts and methods, quantitative data and formulae. Wiley.

[bibr105-2041669520958431] Zhang X. Funt B. Mirzaei H. (2016). Metamer mismatching in practice versus theory. Journal of the Optical Society of America A, 33(3), A238–A247. 10.1364/JOSAA.33.00A238 26974929

